# A Universal Pharmacological-Based List of Drugs with Anticholinergic Activity

**DOI:** 10.3390/pharmaceutics15010230

**Published:** 2023-01-10

**Authors:** Marta Lavrador, Ana C. Cabral, Manuel T. Veríssimo, Fernando Fernandez-Llimos, Isabel V. Figueiredo, M. Margarida Castel-Branco

**Affiliations:** 1 Pharmacology and Pharmaceutical Care Laboratory, Faculty of Pharmacy, University of Coimbra, 3000-548 Coimbra, Portugal; 2 Coimbra Institute for Clinical and Biomedical Research (iCBR), 3000-548 Coimbra, Portugal; 3 Faculty of Medicine, University of Coimbra, 3000-548 Coimbra, Portugal; 4 Laboratory of Pharmacology, Department of Drug Sciences, Faculty of Pharmacy, University of Porto, 4050-313 Porto, Portugal; 5 Center for Health Technology and Services Research (CINTESIS), University of Porto, 4200-450 Porto, Portugal

**Keywords:** aged, anticholinergic burden, receptors, muscarinic, cholinergic antagonists, clinical practice

## Abstract

Anticholinergic burden tools have relevant pharmacological gaps that may explain their limited predictive ability for clinical outcomes. The aim of this study was to provide a universal pharmacological-based list of drugs with their documented affinity for muscarinic receptors. A comprehensive literature review was performed to identify the anticholinergic burden tools. Drugs included in these instruments were searched in four pharmacological databases, and the investigation was supplemented with PubMed. The evidence regarding the potential antagonism of the five muscarinic receptors of each drug was assessed. The proportion of drugs included in the tools with an affinity for muscarinic receptors was evaluated. A universal list of drugs with anticholinergic activity was developed based on their documented affinity for the different subtypes of muscarinic receptors and their ability to cross the blood-brain barrier. A total of 23 tools were identified, including 304 different drugs. Only 48.68%, 47.70%, 48.03%, 43.75%, and 42.76% of the drugs had an affinity to the M1, M2, M3, M4, and M5 receptor, respectively, reported in any pharmacological database. The proportion of drugs with confirmed antagonism varied among the tools (36.8% to 100%). A universal pharmacological-based list of 133 drugs is presented. It should be further validated in different clinical settings.

## 1. Introduction

Drugs with anticholinergic effects are associated with the development of peripheral and central adverse effects, which is particularly relevant for older people, because of age-related physiological changes [1,2,3]. However, they remain poorly recognized concerning their anticholinergic properties, despite being included in the explicit criteria of potential inappropriate medications for elderly people. Anticholinergic burden is defined as the cumulative effect of using one or more medicines with anticholinergic effects. Anticholinergic burden scales and indices are a specific type of explicit criteria to be used in medication reviews in older patients [4,5,6].

A high number of anticholinergic burden tools have already been identified by several systematic reviews [7,8,9,10,11,12,13,14,15]. None of the existing tools have been considered as an option for universal use. The existing evidence suggests that the association among the different scales and indices and the development of negative clinical outcomes is very inconsistent. As a consequence, it is not possible to infer which tool has a better predictive ability for the different outcomes [8,15].

Drugs with anticholinergic effects refer to drugs that bind exclusively to muscarinic receptors (M1, M2, M3, M4, and M5), and so the antagonism of these receptors corresponds to their primary mechanism of action (e.g., oxybutynin, trihexyphenidyl, and ipratropium bromide), and to drugs whose anticholinergic activity is not connected with their primary therapeutic purpose and mechanism of action (e.g., antidepressants, antipsychotics, and antihistamines). The most appropriate term for this drug class should be “muscarinic receptor antagonists”, but the literature mostly presents the term “anticholinergic drugs” or as drugs with anticholinergic activity [16]. In both cases, blockades of nicotinic receptor sites attributed to these drugs is negligible [17].

Most drugs with anticholinergic activity are nonselective for receptor binding and are not tissue-selective. The distribution of muscarinic receptors across many physiological systems leads to a wide range of peripheral (e.g., dry mouth, dry eyes, urinary retention, constipation and tachycardia) and central (e.g., cognitive impairment, delirium, and confusion) adverse effects [18].

We have previously developed a narrative literature review that aimed to assess the rationale and pharmacological basis of the published anticholinergic burden tools [18]. This work has shown that the tools present several gaps concerning their pharmacological basis. Many of them were based on the laboratory assay for serum anticholinergic activity and on subjective expert opinions, both with important limitations. Additionally, the majority of anticholinergic burden tools do not consider the dose of the included anticholinergic drugs; all of them adopt linear models for the cumulative effects (neglecting the possibility of synergistic or antagonistic effects of drugs); almost all ignore the pharmacological characteristics of the different muscarinic receptors (pharmacodynamic limitation) and their distribution across the human body (pharmacokinetic limitation); and they do not consider the frailty and individual characteristics of patients [18].

If anticholinergic burden tools do not have a robust pharmacological basis, they cannot truly assist health care professionals and be valid tools to be used in clinical practice. It is important that these tools create alerts with clinical relevance to avoid situations of alert fatigue that may compromise patient safety. Pharmacological mechanisms are complex and sometimes difficult to interpret. The first important step to better understand the reason why a specific drug is included in a given anticholinergic tool, is to find out the underlying pharmacological mechanism. The competitive antagonism of muscarinic receptors can be considered one basilar mechanism for a drug to be included in the tools.

Therefore, the aim of this research is to analyze the pharmacological basis that supports the inclusion of drugs into the different anticholinergic burden instruments, taking into consideration the evidence about their antagonism of the five subtypes of muscarinic receptors, and to provide a universal pharmacological-based list of drugs with a documented affinity for muscarinic receptors.

## 2. Materials and Methods

### 2.1. Drugs Included in Anticholinergic Burden Tools

We first considered previous systematic reviews to identify the already-published anticholinergic burden tools [7,8,11,14,15]. Anticholinergic burden tools that presented a list of drugs with anticholinergic effects were included. Anticholinergic burden indices that were only based on an equation to estimate anticholinergic burden without a defined list of anticholinergic drugs were excluded (Drug Burden Index (DBI) [19]; Drug Burden Index–World Health Organization (DBI-WHO) [20]; and nonlinear pharmacological binding model [21]).

All drugs included in the mentioned anticholinergic burden tools were compiled and classified according to the Anatomical Therapeutic Chemical (ATC) classification.

### 2.2. Pharmacological Databases

Drugs identified in the included anticholinergic burden tools were then searched in four different pharmacological databases—DrugBank Online; International Union of Basic and Clinical Pharmacology (IUPHAR)/British Pharmacological Society (BPS) Guide to PHARMACOLOGY; Inxight Drugs; and the Psychoactive Drug Screening Program K_i_ database—to assess the evidence about the potential antagonism of the muscarinic receptors of each drug.

We assessed the interaction of all drugs included in anticholinergic burden tools with muscarinic receptors, which are defined as follows: muscarinic acetylcholine receptor (no specific subtype); muscarinic acetylcholine receptor M1; muscarinic acetylcholine receptor M2; muscarinic acetylcholine receptor M3; muscarinic acetylcholine receptor M4; and muscarinic acetylcholine receptor M5. Priority was always given to the data that discriminated binding for each of the receptor’s subtypes. However, when the only information available was the family of muscarinic receptors, that was the data considered.

#### 2.2.1. DrugBank Online

DrugBank Online (https://go.drugbank.com/ accessed on 15 June 2022) is a comprehensive, free-to-access, online database that includes information about drugs and drug targets. It is based on bioinformatics and chemoinformatics principles, and it compiles detailed information about drugs (in particular their chemical structures, pharmacological characteristics, and pharmaceutical aspects) and their pharmacological targets (specifically their sequences, structures, and signaling pathways) [22].

Considering the aim of the present work, we focused our research on the antagonism of the five muscarinic receptors of the drugs identified in the previous step. This information was obtained in the “pharmacological targets and off-target interactions” section of DrugBank Online. Indeed, this section presents the different targets with which a particular drug interacts, presenting, in most cases, the type of action on that target, namely, whether the drug is an agonist, partial agonist, antagonist, inhibitor, or simply binds to that receptor. For some targets, the action is classified as “unknown”. For some drugs, in addition to identifying whether the interaction with muscarinic receptors does exist, DrugBank Online characterizes the binding properties of the interaction. Therefore, for some drugs, parameters related to their affinity for muscarinic receptors were available and were assessed: the equilibrium dissociation constant (K_i_) or pK_i_ (the negative logarithm to base 10 of the K_i_), determined in inhibition assays, or the equilibrium dissociation constant (K_d_) or pK_d_ (the negative logarithm to base 10 of the Kd), determined directly in a binding assay using a labeled form of the ligand. In some cases, parameters related to the potency of the drugs regarding their binding to muscarinic receptors were also accessible, namely, through the IC_50_ results.

#### 2.2.2. International Union of Basic and Clinical Pharmacology (IUPHAR)/British Pharmacological Society (BPS) Guide to PHARMACOLOGY

The IUPHAR/BPS Guide to Pharmacology (https://www.guidetopharmacology.org/ accessed on 25 June 2022) is an expert-based resource of ligand–activity–target relationships mostly derived from high-quality pharmacological and medicinal chemistry literature [23]. This database intends to be a unique portal that incorporates pharmacological information and whose main objective is to provide an easily searchable platform with quantitative information about drug targets, prescription medicines, and experimental drugs that act on these targets. Information about the potential antagonism of the muscarinic receptors of each of the drugs under analysis was obtained from the section “biological activity”. Additionally, whenever available, data characterizing drug binding to the 5 muscarinic receptor subtypes were assessed (IC_50_, K_d_, K_i_, pA_2_, pK_d_, and pK_i_).

#### 2.2.3. Psychoactive Drug Screening Program Ki Database (PDSP Ki Database)

The Psychoactive Drug Screening Program Ki database (https://pdsp.unc.edu/databases/kidb.php accessed on 2 July 2022) is a public resource that provides increasing information about the interaction of drugs with multiple molecular targets. This database corresponds to a platform of Ki values, obtained either through previously published data or internal research. On this particular platform, we enter the name of the drug to be investigated in the “test ligand” field. From this research, we obtained all of the receptors with which that drug interacts and the corresponding Ki value (in nM). When more than one Ki value was provided for each receptor subtype, its average value was calculated.

#### 2.2.4. Inxight Drugs

The Inxight Drugs database (https://drugs.ncats.io/ accessed on 10 July 2022) incorporates a large amount of data concerning approved and experimental drugs. The available data come from information from the FDA and private companies, providing information that ranges from the commercialization and regulatory status of a given drug to information about its biological activity and clinical use, among others. Similarly, each drug previously identified in anticholinergic burden scales and indices was assessed individually, with respect to the potential antagonism for the 5 muscarinic receptors. These data were obtained in the “biological activity” section with identification, whenever available, of the parameters that characterize the binding affinity (K_i_, pK_i_, K_d_, pK_d_, IC_50_, and EC_50_).

#### 2.2.5. PubMed

In addition to the investigation in the mentioned pharmacological databases, we performed a search in PubMed to maximize the information obtained for each of the drugs under analysis, specifically data related to the parameters that characterize the binding affinity for muscarinic receptors.

The following search equation was applied: (ki[TIAB] OR pki[TIAB] OR ic50[TIAB] OR pic50[TIAB] OR kd[TIAB] OR pkd[TIAB] OR pa2[TIAB]) AND (muscarin*[TIAB] OR antimuscarin*[TIAB] OR cholinerg*[TIAB] OR anticholine*[TIAB] OR acetilcholi*[TIAB] OR ach[TIAB]) AND DRUG[TIAB]

The proportion of drugs included in the anticholinergic burden tools with affinity for muscarinic receptors according to each database was evaluated.

### 2.3. Development of a Universal List of Drugs with Anticholinergic Activity

#### 2.3.1. Affinity for Muscarinic Receptors

Considering the results obtained in the previous sections, a new list of drugs with anticholinergic activity is presented, taking into account their documented affinity for the different subtypes of muscarinic receptors.

The following criteria were considered to classify anticholinergic activity of drugs, based on previous work published by Bishara et al. [24]:

- Drugs with pKi < 5.00—excluded
- Drugs with 5.00 ≤ pKi ≤ 5.99—classification +
- Drugs with 6.00 ≤ pKi ≤ 7.00—classification ++
- Drugs with pKi > 7—classification +++

Regarding the drugs for whom the results of the pKi parameter were not identified, the remaining measures (pKd, pIC50, or pA2) were considered, adopting the same cut-offs that were described for the pKi. For some drugs, drug selectivity to the receptor subtypes was not available, so general muscarinic binding was considered. Regarding the drugs for which affinity for muscarinic receptors was recognized in any database but no experimental measure of ligand action was identified, the symbol “✓” was placed. The symbol “0” was assigned to receptor subtypes for which no specific affinity was found when it was identified in other subtypes. It is important to highlight that the classification presented is not intended to provide drug scores, but rather a qualitative classification based on objective pharmacological information.

#### 2.3.2. Ability to Cross BBB

In addition, information regarding the ability of drugs to cross the BBB was also identified. This information can also be found in Inxight:Drugs and DrugBank databases. The former provides information on whether the drug crosses (+) the BBB or not (−). DrugBank presents this information based on a predictive software—admetSAR (absorption, distribution, metabolism, excretion, toxicity structure–activity relationship database). Thus, for each drug with affinity for at least one muscarinic receptor, information about the ability to cross the BBB was provided by Inxight:Drugs database. For drugs that did not have this information available, DrugBank was consulted.

## 3. Results

### 3.1. Drugs included in Anticholinergic Burden Tools

A total of 23 anticholinergic burden scales and indices with a predefined list of drugs with anticholinergic effects were identified: Anticholinergic Drug Scale (ADS) [25]; Anticholinergic Risk Scale (ARS) [26]; Anticholinergic Cognitive Burden Scale (ACB) [27]; Anticholinergic Activity Scale (AAS) [28]; Anticholinergic Burden Classification (ABC) [29]; Anticholinergic Loading Scale (ACL) [30]; Cancelli’s Anticholinergic Burden Scale [31]; Chew’s list [32]; Clinical Index and Pharmacological Index [33]; Clinician-rated Anticholinergic Score (CrAS) [34]; Summers’ Drug Risk Number (DRN) [35]; Muscarinic Acetylcholinergic Receptor ANTagonist Exposure Scale (MARANTE scale) [36]; Anticholinergic Effect on Cognition (AEC) [24]; Anticholinergic Burden Score for German prescribers [37]; Korean Anticholinergic Burden Scale [38]; Anticholinergic Impregnation Scale (AIS) [39]; Brazilian’s scale [40]; Cao’s scale [41]; Drug Delirium Scale (DDS) [42]; Deliriogenic Risk Scale (DRS) [43]; Anticholinergic Toxicity Score (ATS) [44]; Salahudeen’s composite rating scale [11]; and Durán’s list [7].

A total of 304 drugs with anticholinergic effects were identified by at least one of the instruments included. The scores assigned to each drug vary according to the anticholinergic burden scale or index. Detailed information about all of the drugs identified and the different scores attributed to them according to each anticholinergic burden scale or index is presented in supplementary materials (Table S1).

**Figure 1:**
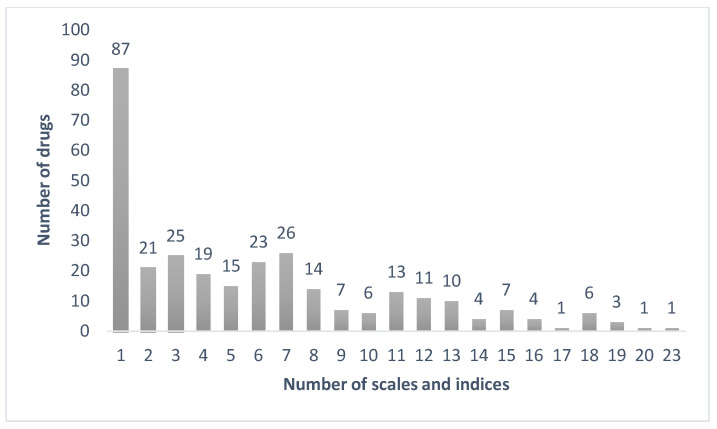
Distribution of the number of drugs according to the number of anticholinergic burden tools.

Only amitriptyline was identified by all 23 anticholinergic burden scales and indices, followed by imipramine (identified by 20 instruments) and diphenhydramine, nortriptyline, and oxybutynin (identified by 19 instruments); conversely, a total of 87 drugs were only identified by one scale or index (Figure 1). Regarding the drugs that were only identified by one scale or index, 28 (32.18%) were only identified by the Korean Anticholinergic Burden Scale; 26 (29.89%) by Summers’ DRN; 9 (10.34%) by DDS; 7 (8.05%) by DRS; 3 (3.45%) by AEC, AIS, and German’s scale; 2 by Cancelli’s scale, Brazilian’s scale, and Cao’s scale; and 1 by Chew’s list and Minzenberg’s scale.

### 3.2. Database Drug Coverage

Of the 304 drugs identified by at least one anticholinergic burden tool, 22 were not present in DrugBank Online; 49 drugs were not identified in the IUPHAR/BPS Guide to Pharmacology database; 139 drugs were not included in the PDSP Ki database; and only five drugs were not identified by Inxight Drugs. Table 1 compiles the results obtained in each database concerning the reported affinity of each drug toward each muscarinic receptor subtype. The supplementary material (Table S2) presents detailed information about the drugs identified by each of the pharmacological databases. Using the PubMed research, we identified a total of 125 drugs with an affinity for at least one muscarinic receptor subtype. Overall, we obtained the following results for the 304 drugs regarding the affinity for muscarinic receptors, according to at least one database or PubMed:

- Muscarinic acetylcholine receptor M1—148 drugs (48.68%)
- Muscarinic acetylcholine receptor M2—145 drugs (47.7%)
- Muscarinic acetylcholine receptor M3—146 drugs (48.03%)
- Muscarinic acetylcholine receptor M4—133 drugs (43.75%)
- Muscarinic acetylcholine receptor M5—130 drugs (42.76%)

### 3.3. Anticholinergic Burden Tools and Reported Affinity for Muscarinic Receptors

Considering all the data obtained in the previous steps, Table 2 presents an overview of the number of drugs that have reported affinity for muscarinic receptors, relating this information with the number of anticholinergic burden tools in which drugs are included. Additionally, it shows the number of drugs with no reported affinity for any muscarinic receptor subtypes. It is important to note that muscarinic antagonists lack selectivity, meaning that, in most cases, the drugs that show affinity for muscarinic receptors bind to all muscarinic receptor subtypes.

Table 3 shows the proportion of drugs that have reported an affinity for each of the muscarinic receptor subtypes as a function of the total number of drugs that each anticholinergic burden scale or index includes. The proportion of drugs with antagonism reported in the databases varied among the instruments, with the highest (100%) toward M1, M2, and M3 in Minzenberg’s scale (28 drugs), 92.6% toward M1, M2, and M3 in Cao’s scale (27 drugs), and 85.0% toward M1 and M3 in AEC (60 drugs) and the lowest (36.8%) toward M4 in DRS (106 drugs), 41.3% toward M4 in Summers’ DRN (63 drugs), and 41.9% toward M5 in ADS (117 drugs).

### 3.4. Universal List of Drugs with Anticholinergic Activity

A total of 148 drugs had reported an affinity for at least one muscarinic receptor subtype. From those, 15 drugs were excluded, because they had pKi values < 5 (Table 4).

The final proposal of a universal list of drugs with documented anticholinergic activity, based on objective pharmacological data, is presented in Table 5. The list is presented according to the ATC classification and considers the five muscarinic receptor subtypes. The ability of each drug to cross the BBB is also present, except for 14 drugs (information not available in databases).

## 4. Discussion

The present manuscript shows that less than half of the drugs included in the anticholinergic burden tools have demonstrated an antagonism of muscarinic receptors. Our results may explain the low predictive power of the anticholinergic burden instruments revealed in previous studies. The analysis performed led to the development of a universal list which only includes the drugs with reported antagonism of muscarinic receptors.

The absence of a universal list of drugs with anticholinergic activity was an important limitation identified in the literature. Many anticholinergic burden tools are country specific, limiting the scope and internationalization of the tools [4,15]. To overcome this gap, we considered all the drugs present in the highest number of anticholinergic burden scales and indices thus far. Each drug was individually assessed through research in four different pharmacological databases, and the investigation was supplemented by a search in PubMed.

Only amitriptyline, a tricyclic antidepressant, was present in all 23 anticholinergic burden tools. Imipramine belongs to the same pharmacological class and was present in 20 instruments, followed by nortriptyline (also a tricyclic antidepressant), diphenhydramine (anti-histaminic H1), and oxybutynin (a drug used to treat overactive bladder). Of these drugs, only oxybutynin has antagonism of muscarinic receptors as its primary mechanism of action. Tricyclic antidepressants are a class with recognized anticholinergic properties and are present in a high number of tools with the maximum score in all. Conversely, the drugs that are only present in one anticholinergic burden tool belong to multiple drug classes. Most of these drugs were identified by the Korean scale [38] and in Summers’ DRN [35]. Regarding KABS, this fact is expected once it represents a market with particular characteristics for drugs that do not have marketing approval in European countries (e.g., oxapium iodide and imidafenacin). However, the fact that one drug is only present in one scale or index does not mean that it is not a drug with anticholinergic effects. Eight of the 28 drugs included in KABS correspond to synthetic anticholinergic drugs, namely, esters with tertiary amino groups (ATC code A03AA), quaternary ammonium compounds (ATC code A03AB), and belladonna derivatives (ATC codes A03BA and A03BB). Other anticholinergic drugs used to treat an overactive bladder, gastritis, or gastroduodenal ulcers are also present in this scale (oxapium iodide, imidaphenacin, and tiquizium).

In contrast, the high number of tools in which a particular drug may appear may be the result of the fact that the scales and indices are often based on previously published tools, which results in perpetuating some drugs on a large number of lists; this does not necessarily mean that there are more reasons to consider a particular drug as anticholinergic. This is highly visible in Durán’s [7] list and Salahudeen’s [11] scale, which are systematic reviews of previously published anticholinergic burden tools. Indeed, only 9 out of 23 tools are not based on previous lists (ABC, ACB, AEC, ARS, ATS, Chew’s list, Minzenberg’s scale, Cancelli’s scale, and Summers’ DRN) [15].

Regarding the data provided by Table 2, we observe that 48.28% of the drugs that are only included in one scale or index have an affinity for M1 and M2 receptors, 47.13% have an affinity for M3, and 44.83% have an affinity for M4 and M5. However, we note that only 33.33% of drugs that are included in 10 scales have reported an affinity for muscarinic receptors, and that only one drug (25%) identified by 14 scales or indices reported antagonism. These results provide evidence of the weaknesses in the pharmacological features of anticholinergic burden tools and show the limitations of most of them, as they are based on subjective expert opinions. Actually, only drugs that are included in 16, 17, 19, 20, and 23 instruments have reported an affinity for all muscarinic receptor subtypes. However, the greatest number of instruments is not synonymous with higher percentages in the reported affinity.

The scores given to drugs can be very different depending on the instrument considered. This may be related to the criteria used in the development of scales. As an example, tiotropium has a score of three on the Brazilian scale [40], but a score of one on the German scale [37]. This drug is a pure antimuscarinic drug. However, it is administered by inhalation with limited systemic absorption, so its potential to cause adverse effects is low. Another relevant example is found with solifenacin. This drug is an antimuscarinic, used for conditions such as an overactive bladder and urinary incontinence. It is included in eight of the anticholinergic burden tools (ACB, AEC, German’s scale, KABS, AIS, Brazilian’s scale, DDS, and Salahudeen’s scale) with maximum scores in all of them except for AEC [24] and DDS [42] (score one). The AEC was developed with the aim of identifying anticholinergic drugs that had cognitive adverse effects, using as a classification criteria not only the affinity for the muscarinic receptors that are present in a higher percentage in the CNS (M1, M2 and M4), but also the information on the ability to cross the blood-brain barrier (BBB) or the data present in the literature on its association with cognitive impairment. Similarly, the DDS was also created with the aim of assessing the drugs associated with a greater risk of developing delirium. Solifenacin is a drug with higher selectivity for the bladder than for the CNS and with a higher affinity for the M3 subtype [46,47]. Therefore, the influence of this drug on the development of central adverse effects is expected to be low; thus, the scores assigned by the AEC and DDS are in line with these concepts. Looking only at the drug score can provide misconceptions about the actual anticholinergic properties of the drug. In fact, the properties will always have to be framed in the scale that assigns a certain score.

Fewer than 50% of drugs have reported an affinity for muscarinic receptors. This may indicate that for some drugs, there is no plausible reason to be included in anticholinergic burden tools. Additionally, many tools are based on the previous results of serum anticholinergic activity (SAA), a laboratory technique with relevant limitations [12,48]. Furthermore, there may be additional mechanisms that motivated the inclusion of such drugs in previous tools, but the fact is, that the validation studies show that the association with anticholinergic adverse effects is scarce.

Table 3 shows the anticholinergic burden tools where the percentage of drugs included with a reported affinity for muscarinic receptors is less than 50%. This happens with ADS, Summers’ DRN, German’s scale, DRS, and Salahudeen’s scale for all receptor subtypes; Chew’s list and CrAS for M4 and M5; and ACL, AIS, and Brazilian’s scale for M2, M4 and M5. The lowest results were found for DRS and Summers’ DRN. The first scale was created to identify deliriogenic drug properties, and the authors may not have only included drugs that have anticholinergic effects. Summers’ DRN is a very old tool with outdated information.

The tools with the highest percentage of drugs with reported antagonism of muscarinic receptors (>70%) were ARS, Minzenberg’s scales, AEC, Cao, and ATS. The classification criteria of ARS and AEC were based on equilibrium dissociation constants for muscarinic receptors (pKi) [24,26]. The ATS was based on a computational model that considers the structure of drugs and their bioactivity for the five muscarinic receptor subtypes through the evaluation of structure–bioactivity relationships [44]. It is the only scale that discriminates the affinity of drugs toward different muscarinic receptor subtypes. The Minzenberg’s scale considered the affinity for brain muscarinic receptors through the evaluation of studies that determined the values of the equilibrium dissociation constant (Kd) to displace 3H-QNB (pharmacological index) binding [33]. Furthermore, it complemented these data with the association between drug use and the development of peripheral adverse effects (clinical index). On Cao’s scale, the authors consulted Mosby’s Drug Consult for the identification of drugs with anticholinergic properties, which included only 27 drugs in total [41]. AEC, ATS, and Cao’s scale have not been validated, but the ARS is one of the most validated anticholinergic tools worldwide [14]. The results of the affinity toward muscarinic receptors obtained for this scale are in line with the literature, which considers the ARS as one of the scales with better predictive ability for adverse clinical outcomes [8,14,15,49].

All of the above underlines the need to create a universal list of drugs with anticholinergic activity that presents objective measurements of the binding affinity of different drugs toward the muscarinic receptor subtypes. Additionally, a list that allows the comparison of drugs from the same pharmacological class regarding their affinities for the different muscarinic receptors and the ability to cross the BBB is missing. The literature shows that most of the anticholinergic burden tools adopt linear models to calculate the anticholinergic burden by adding the scores given to each drug. However, it has been demonstrated that this approach might not be correct, as it underestimates the possibility of synergistic or antagonistic effects of drugs and also neglects the influence of patients’ particular characteristics [8]. Therefore, we preferred to create a universal qualitative tool that differentiates users and non-users of drugs with anticholinergic activity, in order to better guide clinical practice. 

Pharmacological mechanisms are complex. However, this approach can lead to more reliable instruments, as we observed that the anticholinergic burden tools with the highest percentage of drugs with a reported affinity for muscarinic receptors are the ones that showed better results in previous validation studies [14,15]. Additionally, the assessment of pharmacodynamic interactions should also be considered available for new licensed drugs, as it would be very helpful to identify and predict some potential adverse reactions. There may be additional mechanisms, but it is clear that there is a lack of association between the available tools and anticholinergic outcomes. We hypothesize that by withdrawing drugs without the antagonism of muscarinic receptors, we will have a greater connection with clinical outcomes. Collaboration between mechanistic and clinical pharmacology is essential to creating reliable instruments to increase patient safety by reducing anticholinergic adverse outcomes. 

## 5. Conclusions

The currently available anticholinergic burden scales and indices have a great potential to be improved, since their usefulness in clinical practice is limited. A universal list of 133 drugs with anticholinergic activity was created, which presents in a discriminating way, the different affinities of the drugs for the different muscarinic receptor subtypes assessed through objective methods. Further research is needed to validate this new list in different clinical settings and consider both peripheral and central outcomes.

## Figures and Tables

**Table 1:** Percentage of drugs identified by each database regarding their affinity for each of the muscarinic receptor subtypes.

		DrugBank Online (n = 282)	Guide to Pharmacology (n = 255)	PDSP K_i_ Database (n = 165)	Inxight: Drugs (n = 299)
M1	No reported affinity	67.02	80.00	57.58	75.25
	With reported affinity	23.40	8.24	42.42	15.05
M2	No reported affinity	70.57	80.00	57.58	77.59
	With reported affinity	19.86	8.24	42.42	12.71
M3	No reported affinity	70.57	80.39	60.61	75.59
	With reported affinity	19.86	7.84	39.39	14.72
M4	No reported affinity	75.53	81.18	63.64	80.27
	With reported affinity	14.89	7.06	36.36	10.03
M5	No reported affinity	77.30	81.96	64.85	81.27
	With reported affinity	13.12	6.27	35.15	9.03
Muscarinic receptors	With reported affinity	9.57	11.76	7.27	9.7

M1—muscarinic receptor M1; M2—muscarinic receptor M2; M3—muscarinic receptor M3; M4—muscarinic receptor M4; and M5—muscarinic receptor M5.

**Table 2:** Number of drugs with reported affinity for muscarinic receptors and their relationship with the number of anticholinergic burden tools in which they are included.

		Affinity M1		Affinity M2		Affinity M3		Affinity M4		Affinity M5		No Reported Affinity	
Number Tools	Number Drugs	n	%	n	%	n	%	n	%	n	%	n	%
1	87	42	48.28	42	48.28	41	47.13	39	44.83	39	44.83	42	48.28
2	21	7	33.33	7	33.33	7	33.33	5	23.81	5	23.81	14	66.67
3	25	7	28.00	7	28.00	6	24.00	6	24.00	4	16.00	18	72.00
4	19	9	47.37	8	42.11	9	47.37	7	36.84	7	36.84	10	52.63
5	15	3	20.00	4	26.67	3	20.00	2	13.33	3	20.00	11	73.33
6	23	8	34.78	8	34.78	8	34.78	8	34.78	8	34.78	15	65.21
7	26	13	50.00	13	50.00	13	50.00	12	46.15	11	42.31	13	50.00
8	14	8	57.14	7	50.00	8	57.14	7	50.00	7	50.00	6	42.86
9	7	3	42.86	3	42.86	3	42.86	2	28.57	2	28.57	4	57.14
10	6	2	33.33	2	33.33	2	33.33	2	33.33	2	33.33	4	66.67
11	13	8	61.54	8	61.54	8	61.54	8	61.54	7	53.85	5	38.46
12	11	10	90.91	9	81.82	10	90.91	9	81.82	9	81.82	1	9.09
13	10	6	60.00	5	50.00	6	60.00	5	50.00	5	50.00	4	40.00
14	4	1	25.00	1	25.00	1	25.00	1	25.00	1	25.00	3	75.00
15	7	6	85.71	6	85.71	6	85.71	5	71.43	5	71.43	1	14.29
16	4	4	100	4	100	4	100	4	100	4	100	0	-
17	1	1	100	1	100	1	100	1	100	1	100	0	-
18	6	5	83.33	5	83.33	5	83.33	5	83.33	5	83.33	1	16.67
19	3	3	100	3	100	3	100	3	100	3	100	0	-
20	1	1	100	1	100	1	100	1	100	1	100	0	-
23	1	1	100	1	100	1	100	1	100	1	100	0	-

M1—muscarinic receptor M1; M2—muscarinic receptor M2; M3—muscarinic receptor M3; M4—muscarinic receptor M4; and M5—muscarinic receptor M5.

**Table 3:** Proportion of drugs with reported affinity for each of the muscarinic receptor subtypes as a function of the total number of drugs that each anticholinergic burden tool includes.

		Affinity M1		Affinity M2		Affinity M3		Affinity M4		Affinity M5	
Anticholinergic Burden Tool	Number Drugs	n	%	n	%	N	%	n	%	n	%
*ADS*	117	58	49.6	57	48.7	58	49.6	52	44.4	49	41.9
*ARS*	49	38	77.6	38	77.6	38	77.6	36	73.5	35	71.4
*ACB*	99	68	68.7	67	67.7	68	68.7	63	63.6	60	60.6
*AAS*	29	20	69.0	19	65.5	20	69.0	19	65.5	19	65.5
*ABC*	27	18	66.7	18	66.7	17	63.0	17	63.0	17	63.0
*ACL*	49	25	51.0	23	46.9	25	51.0	22	44.9	23	46.9
*Cancelli*	17	10	58.8	10	58.8	10	58.8	9	52.9	9	52.9
*Chew’s list*	39	22	56.4	21	53.8	22	56.4	19	48.7	19	48.7
*Minzenberg (CI e PI)*	28	28	100.0	28	100.0	28	100.0	27	96.4	27	96.4
*CrAS*	59	33	55.9	32	54.2	33	55.9	30	50.8	29	49.2
*Summers’ DRN*	63	27	42.9	29	46.0	28	44.4	26	41.3	27	42.9
*MARANTE*	41	28	68.3	26	63.4	28	68.3	26	63.4	26	63.4
*AEC*	60	51	85.0	50	83.3	51	85.0	48	80.0	46	76.7
*German scale*	151	70	46.4	67	44.4	69	45.7	65	43.0	64	42.4
*KABS*	138	99	71.7	94	68.1	98	71.0	89	64.5	87	63.0
*AIS*	128	66	51.6	64	50.0	66	51.6	61	47.7	61	47.7
*Brazilian scale*	125	66	52.8	63	50.4	66	52.8	61	48.8	61	48.8
*Cao’s scale*	27	25	92.6	25	92.6	25	92.6	24	88.9	22	81.5
*DDS*	96	56	58.3	52	54.2	56	58.3	49	51.0	48	50.0
*DRS*	106	44	41.5	42	39.6	44	41.5	39	36.8	40	37.7
*ATS*	25	21	84.0	21	84.0	21	84.0	20	80.0	19	76.0
*Salahudeen*	192	96	50.0	93	48.4	95	49.5	86	44.8	83	43.2
*Durán*	100	70	70.0	68	68.0	69	69.0	64	64.0	62	62.0

ADS: Anticholinergic Drug Scale; ARS: Anticholinergic Risk Scale; ACB: Anticholinergic Cognitive Burden Scale; AAS: Anticholinergic Activity Scale; ABC: Anticholinergic Burden Classification; ACL: Anticholinergic Loading Scale; CI: Clinical Index; PI: Pharmacological Index; CrAS: Clinician-rated Anticholinergic Score; Summers’ DRN: Summers’ Drug Risk Number; MARANTE: Muscarinic Acetylcholinergic Receptor ANTagonist Exposure Scale; AEC: Anticholinergic Effect on Cognition; KABS: Korean Anticholinergic Burden Scale; AIS: Anticholinergic Impregnation Scale; DDS: Drug Delirium Scale; DRS: Delirogenic Risk Scale; ATS: Anticholinergic Toxicity Score. M1—muscarinic receptor M1; M2—muscarinic receptor M2; M3—muscarinic receptor M3; M4—muscarinic receptor M4; and M5—muscarinic receptor M5. Extracted from [45].

**Table 4:** Drugs excluded from the final list for having pKi values lower than five.

Drug	ATC	M1_pK_i_	M2_pK_i_	M3_pK_i_	M4_pK_i_	M5_pK_i_	Mu_pK_i_
Acetylsalicylic acid	N02BA01		<5			<5	
Amisulpride	N05AL05	<5	<5	<5	<5	<5	
Buproprion	N06AX12	<5	<5	<5	<5	<5	
Celecoxib	M01AH01		<5			<5	
Desvenlafaxine	N06AX23	<5	<5	<5	<5	<5	
Fluvoxamine	N06AB08	<5					<5
Metoclopramide	A03FA01						<5
Molindone	N05AE02	3.55	3.35	<5		<5	
Pramipexole	N04BC05	<5	<5	<5	<5	<5	
Trazodone	N06AX05	<5	<5	<5	<5	<5	
Venlafaxine	N06AX16	<5	<5	<5	<5	<5	4.52
Thiopental	N05CA19			4.12			
Cimetidine	A02BA01						4.14
Ranitidine	A02BA02						3.92
Terfenadine	R06AX12						4.92

pK_i_—the negative logarithm to base 10 of the K_i._ M1—muscarinic receptor M1; M2—muscarinic receptor M2; M3—muscarinic receptor M3; M4—muscarinic receptor M4; M5—muscarinic receptor M5; and Mu—muscarinic acetylcholine receptor (no specific subtype).

**Table 5:** Universal list of drugs with documented anticholinergic activity, based on objective pharmacological data, according to ATC classification, and considering the five muscarinic receptor subtypes.

ATC CLASSIFICATION	Drug	M1	M2	M3	M4	M5	Mu	BBB
A03A. DRUGS FOR FUNCTIONAL GASTROINTESTINAL DISORDERS								
A03AA. Synthetic anticholinergics, esters with tertiary amino group								
A03AA04	Mebeverine	✓	✓	✓	✓	✓		?
A03AA05	Trimebutine	✓	✓	✓	✓	✓		+
A03AA07	Dicyclomine	+++	++	+++	+++	0		+
A03AA09	Difemerine	✓	✓	✓	✓	✓		?
A03AB. Synthetic anticholinergics, quaternary ammonium compounds								
A03AB05	Propantheline	+++	+++	+++	+++	0		-
A03AB06	Octylonium bromide	+++	++	++	++	0		-
A03AB17	Tiemonium	✓	✓	✓	✓	✓		?
A03AB19	Timepidium	+++	+++	+++	+++	+++		-
A03AX. Other drugs for functional gastrointestinal disorders								
A03AX14	Valethamate bromide	✓	✓	✓	✓	✓		?
A03B. BELLADONNA AND DERIVATIVES, PLAIN								
A03BA. Belladonna alkaloids, tertiary amines								
A03BA01	Atropine	+++	+++	+++	+++	+++		+
A03BA03	Hyoscyamine	+++	+++	+++	+++	+++		+
A03BA04	Belladona	✓	✓	✓	✓	✓		+
A03BB. Belladonna alkaloids, semisynthetic, and quaternary ammonium compounds								
A03BB01	Butylscopolamine	0	++	++	0	0		-
A03BB05	Cimetropium	✓	✓	✓	✓	✓		?
A03BB06	Homatropine	+++	++	+++	+++	+++		-
A03C. ANTISPASMODICS IN COMBINATION WITH PSYCHOLEPTICS								
A03CA. Synthetic anticholinergic agents in combination with psycholeptics								
A03CA02	Clidinium	0	0	+++	0	0		+
A04A. ANTIEMETICS AND ANTINAUSEANTS								
A04AD. Other antiemetics								
A04AD01	Scopolamine	+++	+++	+++	+++	+++		+
C01B. ANTIARRHYTHMICS, CLASS I AND III								
C01BA. Antiarrhythmics, class Ia								
C01BA01	Quinidine	0	+++	0	0	0		+
C01BA02	Procainamide	0	+++	0	0	0		+
C01BA03	Disopyramide	+	+	0	0	0	+	+
C01BD. Antiarrhythmics, class III								
C01BD01	Amiodarone						+	+
G04B. UROLOGICALS								
G04BD. Drugs for urinary frequency and incontinence								
G04BD01	Emepronium	✓	✓	✓	✓	✓		?
G04BD02	Flavoxate	✓	✓	✓	✓	✓		+
G04BD04	Oxybutynin chloride	+++	+++	+++	+++	+++		+
G04BD06	Propiverine	++	+	++	++	0		?
G04BD07	Tolterodine tartrate	+++	+++	+++	+++	+++		+
G04BD08	Solifenacin	+++	++	+++	+++	+++		+
G04BD09	Trospium chloride	+++	+++	+++	+++	+++		-
G04BD10	Darifenacin	+++	+++	+++	+++	+++		+
G04BD11	Fesoterodine	+++	++	+++	+++	+++		+
-	Imidafenacin	+++	+++	+++	+++	+++		+
M01A. ANTIINFLAMMATORY AND ANTIRHEUMATIC PRODUCTS, NON-STEROIDS								
M01AH. Coxibs								
M01AH05	Etoricoxib	+	0	0	0	0		+
M03A. MUSCLE RELAXANTS, PERIPHERALLY ACTING AGENTS								
M03AC. Other quaternary ammonium compounds								
M03AC01	Pancuronium	++	+++	++	++	+		-
M03B. MUSCLE RELAXANTS, CENTRALLY ACTING AGENTS								
M03BX. Other centrally acting agents								
M03BX03	Pridinol							+
M03BX08	Cyclobenzaprine	+++	+++	+++	0	0		+
N02A. OPIOIDS								
N02AB. Phenylpiperidine derivatives								
N02AB02	Pethidine						++	+
N02AB03	Fentanyl						+	+
N02AX. Other opioids								
N02AX02	Tramadol	0	0	++	0	0		+
N04A. ANTICHOLINERGIC AGENTS								
N04AA. Tertiary amines								
N04AA01	Trihexyphenidyl	+++	+++	+++	+++	+++		+
N04AA02	Biperiden	+++	+++	+++	+++	0		+
N04AA04	Procyclidine	+++	+++	+++	+++	+++		+
N04AA12	Tropatepine	✓	✓	✓	✓	✓		?
N04AB. Ethers chemically close to antihistamines								
N04AB02	Orphenadrine	+++	++	++	++	++		+
N04AC. Ethers of tropine or tropine derivatives								
N04AC01	Benzatropine	+++	+++	+++	+++	+++		+
N05A. ANTIPSYCHOTICS								
N05AA. Phenothiazines with aliphatic side-chain								
N05AA01	Chlorpromazine	+++	++	+++	+++	+++		+
N05AA02	Levomepromazine						++	+
N05AA03	Promazine	+++	++	0	0	0		+
N05AA04	Acepromazine							+
N05AA06	Cyamemazine	+++	+++	+++	+++	+++		+
N05AB. Phenothiazines with piperazine structure								
N05AB02	Fluphenazine	+	+	+	+	++		+
N05AB03	Perphenazine	+	+	+	0	0		+
N05AB04	Prochlorperazine	++	+	0	0	0		+
N05AB06	Trifluoperazine	+	+	++	0	0		+
N05AC. Phenothiazines with piperidine structure								
N05AC01	Periciazine	✓	✓	✓	✓	✓		+
N05AC02	Thioridazine	+++	++	+++	++	+++	++	+
N05AC03	Mesoridazine	+++	+++	+++	+++	+++	+++	+
N05AC04	Pipotiazine	✓	✓	✓	✓	✓		+
N05AD. Butyrophenone derivatives								
N05AD01	Haloperidol	+	+	0	+	+		+
N05AD06	Bromperidol	+	+	+	+	+		?
N05AE. Indole derivatives								
N05AE03	Sertindole	++	0	+	0	0		+
N05AE04	Ziprasidone	+	+	+	+	+		+
N05AF. Thioxanthene derivatives								
N05AF01	Flupentixol	✓	✓	✓	✓	✓		+
N05AF03	Chlorprothixene	+++	+++	+++	+++	+++		+
N05AF04	Tiotixene	+	+	+	0	0		+
N05AG. Diphenylbutylpiperidine derivatives								
N05AG02	Pimozide			+			+	+
N05AH. Diazepines, oxazepines, thiazepines, and oxepines								
N05AH01	Loxapine	++	++	++	++	++		+
N05AH02	Clozapine	+++	+++	+++	+++	+++		+
N05AH03	Olanzapine	+++	++	+++	++	+++		+
N05AH04	Quetiapine	++	++	+	++	+		+
N05AH05	Asenapine	+	0	0	+	0		+
N05AX. Other antipsychotics								
N05AX08	Risperidone	0	0	0	+	0		+
N05AX11	Zotepine	+++	++	+++	+++	++	++	+
N05AX12	Aripiprazol	+	+	+	+	+		+
N05AX14	Iloperidone	+	+	+	+	+		+
N05	Blonanserin						++	+
N05B. ANXIOLYTICS								
N05BB. Diphenylmethane derivatives								
N05BB01	Hydroxyzine						+	+
N06A. ANTIDEPRESSANTS								
N06AA. Non-selective monoamine reuptake inhibitors								
N06AA01	Desipramine	++	++	++	++	++		+
N06AA02	Imipramine	+++	+++	+++	++	+++		+
N06AA04	Clomipramine						+++	+
N06AA05	Opipramol	✓	✓	✓	✓	✓		?
N06AA06	Trimipramine						+++	+
N06AA07	Lofepramine	+++	++	++	++	++		+
N06AA09	Amitriptyline	+++	+++	+++	+++	+++		+
N06AA10	Nortriptyline	+++	++	+++	+++	+++		+
N06AA11	Protriptyline						+++	+
N06AA12	Doxepin	+++	++	+++	+++	+++		+
N06AA16	Dosulepin	+++	++	+++	+++	+++		+
N06AA17	Amoxapine						++	+
N06AA21	Maprotiline	✓	✓	✓	✓	✓		+
N06AB. Selective serotonin reuptake inhibitors								
N06AB03	Fluoxetine	++	+	++	+	+		+
N06AB04	Citalopram	+	0	+	0	0		+
N06AB05	Paroxetine	++	++	+++	++	++		+
N06AB06	Sertraline	++	+	+	+	+		+
N06AB10	Escitalopram	+	0	+	0	0		+
N06AG. Monoamine oxidase A inhibitors								
N06AG02	Moclobemide	✓	✓	✓	✓	✓		+
N06AX. Other antidepressants								
N06AX06	Nefazodone						+	+
N06AX11	Mirtazapine			++			+	+
N06AX21	Duloxetine	++	++	+	0	0		+
N07C. ANTIVERTIGO PREPARATIONS								
N07CA. Antivertigo preparations								
N07C	Difenidol	++	+	++	++	+		+
N07CA02	Cinnarizine						++	+
R03B. OTHER DRUGS FOR OBSTRUCTIVE AIRWAY DISEASES, INHALANTS								
R03BB. Anticholinergics								
R03BB01	Ipratropium	+++	+++	+++	+++	+++		+
R03BB02	Oxitropium bromide	+++	+++	+++	0	0		?
R03BB04	Tiotropium	+++	+++	+++	+++	+++		-
R03BB05	Aclidinium	+++	+++	+++	+++	+++		-
R03BB06/A03AB02	Glycopyrronium/Glycopyrrolate	+++	+++	+++	+++	+++		-
R05D. COUGH SUPPRESSANTS, EXCL. COMBINATIONS WITH EXPECTORANTS								
R05DB. Other cough suppressants								
R05DB21	Cloperastine	✓	✓	✓	✓	✓		-
R06A. ANTIHISTAMINES FOR SYSTEMIC USE								
R06AA. Aminoalkyl ethers								
R06AA02	Diphenhydramine	++	++	++	++	++	++	+
R06AA04	Clemastine						+++	+
R06AA07	Piprinhydrinate						+++	+
R06AA08	Carbinoxamine						++	+
R06AA09	Doxylamine	✓	✓	✓	✓	✓		+
R06AA11	Dimenhydrinate						++	+
R06AB. Substituted alkylamines								
R06AB01	Brompheniramine	✓	✓	✓	✓	✓		+
R06AB02	Dexchlorpheniramine						++	+
R06AB03	Dimetindene	++	++	++	++	+		-
R06AB06	Dexbrompheniramine	✓	✓	✓	✓	✓		+
R06AD. Phenothiazine derivatives								
R06AD01	Alimemazine						+++	-
R06AD02	Promethazine						+++	+
R06AD07	Mequitazine	+++	+++	+++	+++	+++		+
R06AE. Piperazine derivatives								
R06AE01	Buclizine	✓	✓	✓	✓	✓		+
R06AE05	Meclizine						+	+
R06AX. Other antihistamines for systemic use								
R06AX02	Cyproheptadine	+++	+++	+++	+++	+++		+
R06AX07	Triprolidine						++	+
R06AX11	Astemizole						+	-
R06AX13	Loratadine	✓	✓	✓	✓	✓		-
R06AX17	Ketotifen	++	++	++	0	0		+
S01F. MYDRIATICS AND CYCLOPLEGICS								
S01FA. Anticholinergics								
S01FA01	Atropine Sulfate ophthalmic	+++	+++	+++	+++	+++		+
S01FA04	Cyclopentolate ophthalmic	+++	+++	+++	+++	0		+
S01FA06	Tropicamide ophthalmic	+++	+++	+++	+++	++		+
S01G. DECONGESTANTS AND ANTIALLERGICS								
S01GX. Other antiallergics								
S01GX08	Ketotifen ophthalmic	++	++	++	0	0		+
DRUGS WITHOUT ATC								
-	Homochlorcyclizine						+++	?
-	Oxapium iodide	✓	✓	✓	✓	✓		?
-	Tiquizium	+++	+++	+++	+++	+++		?

M1—muscarinic receptor M1; M2—muscarinic receptor M2; M3—muscarinic receptor M3; M4—muscarinic receptor M4; and M5—muscarinic receptor M5. Mu: muscarinic acetylcholine receptor (no specific subtype) Classification +**:** 5.00 ≤ pK_i_ ≤ 5.99; 5.00 ≤ pK_d_ ≤ 5.99; 5.00 ≤ pIC_50_ ≤ 5.99; 5.00 ≤ pA_2_ ≤ 5.99. Classification ++**:** 6.00 ≤ pK_i_ ≤ 7.00; 6.00 ≤ pK_d_≤ 7.00; 6.00 ≤ pIC_50_ ≤ 7.00; 6.00 ≤ pA_2_ ≤ 7.00. Classification +++: pK_i_ > 7; pK_d_ > 7; pIC_50_ > 7; pA_2_ > 7. ✓: reported affinity, but no experimental measure of ligand action identified. 0: no specific affinity was found for a particular receptor subtype. BBB: blood-brain barrier. +: drug crosses BBB. -: drug does not cross BBB. ?: information not available in databases.

## Data Availability

Not applicable.

## Supplementary Materials

The following supporting information can be downloaded at https://www.mdpi.com/article/10.3390/pharmaceutics15010230/s1. Table S1: drugs identified by 23 anticholinergic burden tools and scores assigned by each tool; and Table S2: reported affinity of the 304 drugs for muscarinic receptor subtypes according to each pharmacological database.
