# Supplementary material for: A Universal Pharmacological-Based List of Drugs with Anticholinergic Activity
Source: Pharmaceutics. 2023 Jan 10;15(1):230. doi: 10.3390/pharmaceutics15010230 (PMC9863833; doi:10.3390/pharmaceutics15010230)
Supplement: Supplementary file 1 [file pharmaceutics-15-00230-s001.zip › pharmaceutics-2094031-supplementary/Supplementary Table S1.pdf]

Table S1 - Drugs identified by 23 anticholinergic burden tools and scores assigned by each tool.

Drug	1	2	3	4	5	6	7	8	9	10	11	12	13	14	15	16	17	18	19	20	21	22	23
1: Anticholinergic Drug Scale; 2: Anticholinergic Risk Scale; 3: Anticholinergic Cognitive Burden Scale; 4: Anticholinergic Activity Scale; 5: Anticholinergic Burden Classification; 6: Anticholinergic Loading Scale; 7: Cancelli's Anticholinergic Burden Scale; 8: Chew's list; 9: Clinical Index and Pharmacological Index; 10: Clinician-rated Anticholinergic Score; 11: Summers' Drug Risk Number; 12: Muscarinic Acetylcholinergic Receptor ANTagonist Exposure Scale; 13: Anticholinergic Effect on Cognition; 14: Anticholinergic Burden Score for German prescribers; 15: Korean Anticholinergic Burden Scale; 16: Anticholinergic Impregnation Scale; 17: Brazilian's scale; 18: Cao's scale; 19: Drug Delirium Scale; 20: Delirogenic Risk Scale; 21: Anticholinergic Toxicity Score; 22: Salahudeen's Composite rating scale; 23: Durán's list																							
Aceprometazine					3																	1	2
Acepromazine					3																	1	2
Acetazolamide											2												
Acetylsalicylic acid											2												
Aclidinium														1									
Aciclovir																			1				
Alimemazine			1		2								3		1	1						1	1
Alprazolam	1		1		3	1				1				1	1	1	1		1	1	0.6	1	
Alverine			1		2											1						1	
Amiodarone													1										
Amitriptyline	3	3	3	4	3	3	3	4	1	3	3	2	3	3	3	3	3	1	2	4	5	1	2
Amantadine	1	2	2									1	2	2	2	2	2	1	1	1		1	1
Amphotericin																			1				
Amisulpride															1								
Amoxapine			3		3				1						3	3						1	
Amoxicillin								1												1			
Ampicillin	1													1		1	1			1		1	

Drug	1	2	3	4	5	6	7	8	9	10	11	12	13	14	15	16	17	18	19	20	21	22	23
1: Anticholinergic Drug Scale; 2: Anticholinergic Risk Scale; 3: Anticholinergic Cognitive Burden Scale; 4: Anticholinergic Activity Scale; 5: Anticholinergic Burden Classification; 6: Anticholinergic Loading Scale; 7: Cancelli's Anticholinergic Burden Scale; 8: Chew's list; 9: Clinical Index and Pharmacological Index; 10: Clinician-rated Anticholinergic Score; 11: Summers' Drug Risk Number; 12: Muscarinic Acetylcholinergic Receptor ANTagonist Exposure Scale; 13: Anticholinergic Effect on Cognition; 14: Anticholinergic Burden Score for German prescribers; 15: Korean Anticholinergic Burden Scale; 16: Anticholinergic Impregnation Scale; 17: Brazilian's scale; 18: Cao's scale; 19: Drug Delirium Scale; 20: Delirogenic Risk Scale; 21: Anticholinergic Toxicity Score; 22: Salahudeen's Composite rating scale; 23: Durán's list																							
Amobarbital											3												
Aripiprazol			1										1	1	1							1	
Asenapine			1											1								1	
Astemizole																		1					
Atenolol			1							1				1		1	1					1	
Atropine	3	3	3			3		4		3	3		3	3	3	3	3	1	2	4	5	1	2
Azathioprine	1													1		1	1					1	
Baclofen		2								2				1	1	2	2		2	1		1	1
Belladona			2		3					3					3		3					1	2
Benazepril										1				1								1	
Benzatropine	3	3	3	4					1		3		3		3			1	2		5	1	2
Betaxolol										1				1								1	
Biperiden							3		1		3				3	3	3			4			
Bisacodyl						1								1								1	
Blonanserin															1								
Bromazepam																			1	1			
Bromocriptine	1												1	1		1	1					1	1
Brompheniramine	3		3												3	3	3				3,11	1	2
Bromperidol															1								

Drug	1	2	3	4	5	6	7	8	9	10	11	12	13	14	15	16	17	18	19	20	21	22	23
1: Anticholinergic Drug Scale; 2: Anticholinergic Risk Scale; 3: Anticholinergic Cognitive Burden Scale; 4: Anticholinergic Activity Scale; 5: Anticholinergic Burden Classification; 6: Anticholinergic Loading Scale; 7: Cancelli's Anticholinergic Burden Scale; 8: Chew's list; 9: Clinical Index and Pharmacological Index; 10: Clinician-rated Anticholinergic Score; 11: Summers' Drug Risk Number; 12: Muscarinic Acetylcholinergic Receptor ANTagonist Exposure Scale; 13: Anticholinergic Effect on Cognition; 14: Anticholinergic Burden Score for German prescribers; 15: Korean Anticholinergic Burden Scale; 16: Anticholinergic Impregnation Scale; 17: Brazilian's scale; 18: Cao's scale; 19: Drug Delirium Scale; 20: Delirogenic Risk Scale; 21: Anticholinergic Toxicity Score; 22: Salahudeen's Composite rating scale; 23: Durán's list																							
Brotizolam																				1			
Buclizine																	3						
Buprenorphine																			1				
Bupropion			1							1				1	1	1	1		1			1	
Butylscopolamine															3		3						
Captopril	1		1											1		1	1			1		1	
Carbamazepine	2		2							1		1	1	2	1	2	2	1		2		1	1
Carbidopa-levodopa		1				1				1				1		1	1					1	
Carbinoxamine	3		3												3		3				3,25	1	2
Carisoprodol		3															3					1	
Cefamandole	1																					1	
Cefoxitin	1															1	1					1	
Celecoxib						1		1						1						1		1	
Cefalotin	1																					1	
Cefalexin								1												1			
Cetirizine		2	1			2				2		1		1	1	2	2					1	1
Chlordiazepoxide	1									1	3			1	1	1	1		2	1		1	1
Chlorohydrate											2												

Drug	1	2	3	4	5	6	7	8	9	10	11	12	13	14	15	16	17	18	19	20	21	22	23
1: Anticholinergic Drug Scale; 2: Anticholinergic Risk Scale; 3: Anticholinergic Cognitive Burden Scale; 4: Anticholinergic Activity Scale; 5: Anticholinergic Burden Classification; 6: Anticholinergic Loading Scale; 7: Cancelli's Anticholinergic Burden Scale; 8: Chew's list; 9: Clinical Index and Pharmacological Index; 10: Clinician-rated Anticholinergic Score; 11: Summers' Drug Risk Number; 12: Muscarinic Acetylcholinergic Receptor ANTagonist Exposure Scale; 13: Anticholinergic Effect on Cognition; 14: Anticholinergic Burden Score for German prescribers; 15: Korean Anticholinergic Burden Scale; 16: Anticholinergic Impregnation Scale; 17: Brazilian's scale; 18: Cao's scale; 19: Drug Delirium Scale; 20: Delirogenic Risk Scale; 21: Anticholinergic Toxicity Score; 22: Salahudeen's Composite rating scale; 23: Durán's list																							
Chlorpheniramine	3	3	3		3	3				3	3			3	3		3	1	1		5	1	
Chlorphenamine													2			3				4		1	2
Chlorpromazine	3	3	3				3	3	1	3	3		3		3	3	3		1		5	1	2
Chlortalidone	1		1											1		1	1					1	
Chlorprothixene															3								
Chlorzoxazone																	2						
Cimetidine	2	2	1									1		2	2	2	2		1	2		1	1
Cimetropium															3								
Cinnarizine															1								
Citalopram				2		1		2				1	1	1	1	1	1		1	2		1	1
Clemastine	3		3										3	3	3		3	1		4		1	2
Clidinium			1												2				1			1	
Clindamycin	1													1		1				1		1	
Clobazam																				1			
Clomipramine	3		3		3							2	3	3	3	3	3		2	4		1	2
Clonazepam	1					1						1		1	1	1	1		1	1		1	1
Clonidine											1								1	1			
Cloperastine															2								

Drug	1	2	3	4	5	6	7	8	9	10	11	12	13	14	15	16	17	18	19	20	21	22	23
1: Anticholinergic Drug Scale; 2: Anticholinergic Risk Scale; 3: Anticholinergic Cognitive Burden Scale; 4: Anticholinergic Activity Scale; 5: Anticholinergic Burden Classification; 6: Anticholinergic Loading Scale; 7: Cancelli's Anticholinergic Burden Scale; 8: Chew's list; 9: Clinical Index and Pharmacological Index; 10: Clinician-rated Anticholinergic Score; 11: Summers' Drug Risk Number; 12: Muscarinic Acetylcholinergic Receptor ANTagonist Exposure Scale; 13: Anticholinergic Effect on Cognition; 14: Anticholinergic Burden Score for German prescribers; 15: Korean Anticholinergic Burden Scale; 16: Anticholinergic Impregnation Scale; 17: Brazilian's scale; 18: Cao's scale; 19: Drug Delirium Scale; 20: Delirogenic Risk Scale; 21: Anticholinergic Toxicity Score; 22: Salahudeen's Composite rating scale; 23: Durán's list																							
Clorazepate	1		1		3									1	1	1			1	1	1,7	1	
Clotiapine							3																
Clozapine	3	2	3	4			3	4	1			2	3	3	3	3	3			4		1	2
Codeine	1		1		2	1				1	2	1		1	1	1	1		1	1		1	1
Colchicine			1		3											1	1			4		1	
Cortisone	1																		1			1	
Cyamemazine																3							
Cyclobenzaprine	2	2	2							1					2		3		2			1	1
Cyproheptadine	2	3	2			3							3	3	2	3	3	1	1			1	2
Cycloserine	1																					1	
Ciclosporin	1													1		1	1					1	
Darifenacin	3		3											3			3		1			1	2
Desipramine	3	2	3						1	2	3		2				3	1	2			1	2
Desloratadine			1											1	1	3	1					1	
Desvenlafaxine															1				1				
Dexamethasone	1										2			1		1	1		1	1		1	
Dexbrompheniramine															3		3						
Dexchlorpheniramine					3	3									3	3	3					1	2

Drug	1	2	3	4	5	6	7	8	9	10	11	12	13	14	15	16	17	18	19	20	21	22	23
1: Anticholinergic Drug Scale; 2: Anticholinergic Risk Scale; 3: Anticholinergic Cognitive Burden Scale; 4: Anticholinergic Activity Scale; 5: Anticholinergic Burden Classification; 6: Anticholinergic Loading Scale; 7: Cancelli's Anticholinergic Burden Scale; 8: Chew's list; 9: Clinical Index and Pharmacological Index; 10: Clinician-rated Anticholinergic Score; 11: Summers' Drug Risk Number; 12: Muscarinic Acetylcholinergic Receptor ANTagonist Exposure Scale; 13: Anticholinergic Effect on Cognition; 14: Anticholinergic Burden Score for German prescribers; 15: Korean Anticholinergic Burden Scale; 16: Anticholinergic Impregnation Scale; 17: Brazilian's scale; 18: Cao's scale; 19: Drug Delirium Scale; 20: Delirogenic Risk Scale; 21: Anticholinergic Toxicity Score; 22: Salahudeen's Composite rating scale; 23: Durán's list																							
Dextromethorphan										1				1	1							1	
Diazepam	1		1	1		1		1		1	3	1	1	1	1	1	1		2	1	2	1	1
Difenidol															2								
Difemerine															3								
Dicyclomine	3	3	3					4					2		3			1	1		5	1	2
Diphenoxylate								1															
Digitoxin	1			1										1								1	1
Digoxin	1		1	1	3	1	2	1			2			1	1	1	1			1		1	
Diltiazem	1													1		1	1			1		1	
Dimenhydrinate	3		3										2	3	3	3	3		1	4		1	2
Dimetindene														1						1			
Diphenhydramine	3	3	3					3	1	3	3	2	2	3	3	3	3	1	1	3	5	1	2
Dipyridamole	1		1											1			1			1		1	
Disopyramide	2		1										2			2		1				1	1
Disulfiram											2												
Divalproex sodium	1															1						1	
Domperidone						1						1	1	1		1	1					1	1
Donepezil								1												1			

Drug	1	2	3	4	5	6	7	8	9	10	11	12	13	14	15	16	17	18	19	20	21	22	23
1: Anticholinergic Drug Scale; 2: Anticholinergic Risk Scale; 3: Anticholinergic Cognitive Burden Scale; 4: Anticholinergic Activity Scale; 5: Anticholinergic Burden Classification; 6: Anticholinergic Loading Scale; 7: Cancelli's Anticholinergic Burden Scale; 8: Chew's list; 9: Clinical Index and Pharmacological Index; 10: Clinician-rated Anticholinergic Score; 11: Summers' Drug Risk Number; 12: Muscarinic Acetylcholinergic Receptor ANTagonist Exposure Scale; 13: Anticholinergic Effect on Cognition; 14: Anticholinergic Burden Score for German prescribers; 15: Korean Anticholinergic Burden Scale; 16: Anticholinergic Impregnation Scale; 17: Brazilian's scale; 18: Cao's scale; 19: Drug Delirium Scale; 20: Delirogenic Risk Scale; 21: Anticholinergic Toxicity Score; 22: Salahudeen's Composite rating scale; 23: Durán's list																							
Dopamine											2												
Dosulepin						2						1	3			2						1	1
Doxepin	3		3	4		3		4	1	3	3	2	3	3	3	3		1	2	4		1	2
Doxylamine			3											1	3	2	3		1			1	
Duloxetine								1								1			1	1			
Emedastine															1								
Emepronium				4																		1	2
Entacapone		1												1		1	1					1	1
Escitalopram						1		2						1	1		1		1	2		1	
Estazolam	1														1							1	
Ethchlorvynol											2												
Etoricoxib														1									
Famotidine	1													1		1	1					1	
Fentanyl	1		1					1			2	1	1	1	1	1	1		1	1	1,99	1	1
Fesoterodine			3											3	3				1			1	
Fexofenadine						2				2				1		2	1					1	1
Flavoxate	3		3											3	3	3			1			1	2
Fluoxetine	1			2		1		2		1		1	1	1	1	1	1		1	2		1	1
Fluphenazine	1	3				3			1				1	1		3	3		1	1		1	2
Flunitrazepam				1										1	1					1			

Drug	1	2	3	4	5	6	7	8	9	10	11	12	13	14	15	16	17	18	19	20	21	22	23
1: Anticholinergic Drug Scale; 2: Anticholinergic Risk Scale; 3: Anticholinergic Cognitive Burden Scale; 4: Anticholinergic Activity Scale; 5: Anticholinergic Burden Classification; 6: Anticholinergic Loading Scale; 7: Cancelli's Anticholinergic Burden Scale; 8: Chew's list; 9: Clinical Index and Pharmacological Index; 10: Clinician-rated Anticholinergic Score; 11: Summers' Drug Risk Number; 12: Muscarinic Acetylcholinergic Receptor ANTagonist Exposure Scale; 13: Anticholinergic Effect on Cognition; 14: Anticholinergic Burden Score for German prescribers; 15: Korean Anticholinergic Burden Scale; 16: Anticholinergic Impregnation Scale; 17: Brazilian's scale; 18: Cao's scale; 19: Drug Delirium Scale; 20: Delirogenic Risk Scale; 21: Anticholinergic Toxicity Score; 22: Salahudeen's Composite rating scale; 23: Durán's list																							
Flupentixol															1								
Flurazepam	1										3			1	1				2	1		1	
Fluticasone-salmeterol	1																					1	
Fluvoxamine	1		1	2		1								1	1	1	1		1	1		1	1
Furosemide	1		1	1	3		2	1						1	1		1			1		1	
Gabapentin																			1				
Gentamicin	1													1		1	1			1		1	
Glycopyrronium/Glycopyrrolate													1	2			1						
Guaifenesin										1				1	1							1	
Haloperidol		1	1			2			1			1		2	1	1	1				3,78	1	1
Homatropine										3							3					1	2
Homochlorcyclizine															3								
Hydralazine	1		1								1			1	1		1			1		1	
Hydrocodone								1		2					1				1	1		1	1
Hydrocortisone	1		1											1	1	1	1					1	
Hydromorphone																			1				
Hydroxyzine	3	3	3		3						2	2	1	3	3	3	3	1	1	4		1	2

Drug	1	2	3	4	5	6	7	8	9	10	11	12	13	14	15	16	17	18	19	20	21	22	23
1: Anticholinergic Drug Scale; 2: Anticholinergic Risk Scale; 3: Anticholinergic Cognitive Burden Scale; 4: Anticholinergic Activity Scale; 5: Anticholinergic Burden Classification; 6: Anticholinergic Loading Scale; 7: Cancelli's Anticholinergic Burden Scale; 8: Chew's list; 9: Clinical Index and Pharmacological Index; 10: Clinician-rated Anticholinergic Score; 11: Summers' Drug Risk Number; 12: Muscarinic Acetylcholinergic Receptor ANTagonist Exposure Scale; 13: Anticholinergic Effect on Cognition; 14: Anticholinergic Burden Score for German prescribers; 15: Korean Anticholinergic Burden Scale; 16: Anticholinergic Impregnation Scale; 17: Brazilian's scale; 18: Cao's scale; 19: Drug Delirium Scale; 20: Delirogenic Risk Scale; 21: Anticholinergic Toxicity Score; 22: Salahudeen's Composite rating scale; 23: Durán's list																							
Hyoscyamine	3	3	3					4					3		3		3	1			5	1	2
Iloperidone			1										1									1	
Imidafenacin															3								
Imipramine	3	3	3		3	3			1	3	3	2	3	3	3	3	3	1	2	4	5	1	2
Ipratropium				4			2					2		1		3	3					1	2
Isosorbide	1															1	1					1	
Isosorbide dinitrate	1						2				2			1									
Isosorbide mononitrate	1		1											1									
Ketotifen ophthalmic	1																					1	
Ketotifen															1								
Ketorolac										1				1								1	1
Lansoprazole				1				1						1						1			
Levocetirizine			1											1	1		1					1	
Levofloxacin								1												1			
Levomepromazine	2		2		3							2	2	3	2	2	2			4		1	2
Lidocaine											2												
Lithium						1		2					1	1		1	1		1	2		1	1
Lofepramine													3										

Drug	1	2	3	4	5	6	7	8	9	10	11	12	13	14	15	16	17	18	19	20	21	22	23
1: Anticholinergic Drug Scale; 2: Anticholinergic Risk Scale; 3: Anticholinergic Cognitive Burden Scale; 4: Anticholinergic Activity Scale; 5: Anticholinergic Burden Classification; 6: Anticholinergic Loading Scale; 7: Cancelli's Anticholinergic Burden Scale; 8: Chew's list; 9: Clinical Index and Pharmacological Index; 10: Clinician-rated Anticholinergic Score; 11: Summers' Drug Risk Number; 12: Muscarinic Acetylcholinergic Receptor ANTagonist Exposure Scale; 13: Anticholinergic Effect on Cognition; 14: Anticholinergic Burden Score for German prescribers; 15: Korean Anticholinergic Burden Scale; 16: Anticholinergic Impregnation Scale; 17: Brazilian's scale; 18: Cao's scale; 19: Drug Delirium Scale; 20: Delirogenic Risk Scale; 21: Anticholinergic Toxicity Score; 22: Salahudeen's Composite rating scale; 23: Durán's list																							
Loperamide	1	2	1			1				1		1		2	1	2	1				2,45	1	1
Loratadine		2	1			1				1				1	1	2	1	1				1	1
Lorazepam	1													1	1	1	1		1	1		1	
Lormetazepam																				1			
Loxapine	2		2						1					2	2	2			1			1	1
Lumiracoxib						1																1	
Maprotiline					3									2		3	3			4		1	
Mebeverine															1								
Meclizine	3	3	3												3		3	1				1	2
Medazepam																				1			
Mequitazine															3	3							
Mesoridazine									1														
Metaraminol											2												
Metformin						1		1						1						1		1	
Mehadone										2				2		2	2					1	1
Methocarbamol		1	3							1				1	1	1			1			1	1
Methotrexate						1								1								1	
Methohexital											3												
Methyldopa											2												

Drug	1	2	3	4	5	6	7	8	9	10	11	12	13	14	15	16	17	18	19	20	21	22	23
1: Anticholinergic Drug Scale; 2: Anticholinergic Risk Scale; 3: Anticholinergic Cognitive Burden Scale; 4: Anticholinergic Activity Scale; 5: Anticholinergic Burden Classification; 6: Anticholinergic Loading Scale; 7: Cancelli's Anticholinergic Burden Scale; 8: Chew's list; 9: Clinical Index and Pharmacological Index; 10: Clinician-rated Anticholinergic Score; 11: Summers' Drug Risk Number; 12: Muscarinic Acetylcholinergic Receptor ANTagonist Exposure Scale; 13: Anticholinergic Effect on Cognition; 14: Anticholinergic Burden Score for German prescribers; 15: Korean Anticholinergic Burden Scale; 16: Anticholinergic Impregnation Scale; 17: Brazilian's scale; 18: Cao's scale; 19: Drug Delirium Scale; 20: Delirogenic Risk Scale; 21: Anticholinergic Toxicity Score; 22: Salahudeen's Composite rating scale; 23: Durán's list																							
Methylprednisolone	1													1		1	1		1	1		1	
Metoclopramide		1				1				3				1		1	1					1	
Metoprolol			1							1				1		1	1					1	
Midazolam	1													1	1	1	1		1			1	
Mirtazapine		1						2				1	1	1	1	1	1		1	2		1	1
Moclobemide																			1				
Molindone	2		2												2							1	1
Morphine	1		1							1	3	1		1	1	1	1		1	1		1	1
Naratriptan						1								1								1	
Nefopam			2												2							1	
Nefazodone										1												1	1
Nifedipine	1		1				2							1		1	1			1		1	
Nitrazepam																			1	1			
Nitroprusside											2												
Nizatidine	1															1						1	
Nortriptyline	3	2	3	3				3	1	3	3	2	3	3	3	3	3	1	2	3		1	2
Octylonium bromide															3								
Olanzapine	1	2	3	3			2	3	1	1		1	2	2	3	2	3		1	3		1	1

**Drug**	**1**	**2**	**3**	**4**	**5**	**6**	**7**	**8**	**9**	**10**	**11**	**12**	**13**	**14**	**15**	**16**	**17**	**18**	**19**	**20**	**21**	**22**	**23**
1: Anticholinergic Drug Scale; 2: Anticholinergic Risk Scale; 3: Anticholinergic Cognitive Burden Scale; 4: Anticholinergic Activity Scale; 5: Anticholinergic Burden Classification; 6: Anticholinergic Loading Scale; 7: Cancelli's Anticholinergic Burden Scale; 8: Chew's list; 9: Clinical Index and Pharmacological Index; 10: Clinician-rated Anticholinergic Score; 11: Summers' Drug Risk Number; 12: Muscarinic Acetylcholinergic Receptor ANTagonist Exposure Scale; 13: Anticholinergic Effect on Cognition; 14: Anticholinergic Burden Score for German prescribers; 15: Korean Anticholinergic Burden Scale; 16: Anticholinergic Impregnation Scale; 17: Brazilian's scale; 18: Cao's scale; 19: Drug Delirium Scale; 20: Delirogenic Risk Scale; 21: Anticholinergic Toxicity Score; 22: Salahudeen's Composite rating scale; 23: Durán's list																							
Opipramol					3									2						4		1	
Orphenadrine	3		3	4	3								3	3	3		3		1	4	5	1	2
Oxapium iodide															3								
Oxazepam	1					1								1		1			1	1		1	
Oxcarbazepine	2		2											2	2	2	2		1	2		1	1
Oxitropium bromide							2																
Oxybutynin	3	3	3	4	3	2	3	3				2	3	3	3	3	3	1	2	3		1	2
Oxycodone	1					1				1		1		1	1	1	1		1	1		1	1
Paliperidone			1											1	1							1	
Pancuronium	1													1								1	
Paroxetine	1	1	3	3		2		3	1	2		1	2	2	2	2	3		1	3		1	1
Pentazocine											2												
Perphenazine	1	3	3				3		1	2	3		1	1	2	3			1	1	3,9	1	
Pethidine	2		2								3		2	2	2	2	2		2	1		1	1
Phenelzine	1															1			1			1	1
Pheniramine															3								
Phenytoin								1			2									1			
Phenobarbital				1						1	3			1			1		1			1	
Pimozide	2		2						1				2	2	2	2	2		1	2		1	1

Drug	1	2	3	4	5	6	7	8	9	10	11	12	13	14	15	16	17	18	19	20	21	22	23
1: Anticholinergic Drug Scale; 2: Anticholinergic Risk Scale; 3: Anticholinergic Cognitive Burden Scale; 4: Anticholinergic Activity Scale; 5: Anticholinergic Burden Classification; 6: Anticholinergic Loading Scale; 7: Cancelli's Anticholinergic Burden Scale; 8: Chew's list; 9: Clinical Index and Pharmacological Index; 10: Clinician-rated Anticholinergic Score; 11: Summers' Drug Risk Number; 12: Muscarinic Acetylcholinergic Receptor ANTagonist Exposure Scale; 13: Anticholinergic Effect on Cognition; 14: Anticholinergic Burden Score for German prescribers; 15: Korean Anticholinergic Burden Scale; 16: Anticholinergic Impregnation Scale; 17: Brazilian's scale; 18: Cao's scale; 19: Drug Delirium Scale; 20: Delirogenic Risk Scale; 21: Anticholinergic Toxicity Score; 22: Salahudeen's Composite rating scale; 23: Durán's list																							
Piperacillin	1													1		1	1					1	
Pipotiazine																1							
Piprinhydrinate															3								
Pramipexole		1												1		1	1		1			1	
Prazepam																				1			
Prednisolone	1												1	1	1	1	1					1	
Prednisone	1		1								2			1		1	1		1	1		1	
Pregabalin																			1				
Pridinol															3								
Procainamide											2												
Procyclidine	3												3	3	3				2	4		1	2
Prochlorperazine	1	2				2			1	2	3		2			2			1			1	1
Promazine				3			3		1				2									1	1
Prometazine	3	3	3								3	2	3	1		3	3	1	1	4	5	1	2
Propantheline	3		3							2			2					1				1	2
Periciazine																1							
Propiverine			3											3	3							1	
Propoxyphene				1				1		2	2											1	1
Propranolol											2												

Drug	1	2	3	4	5	6	7	8	9	10	11	12	13	14	15	16	17	18	19	20	21	22	23
1: Anticholinergic Drug Scale; 2: Anticholinergic Risk Scale; 3: Anticholinergic Cognitive Burden Scale; 4: Anticholinergic Activity Scale; 5: Anticholinergic Burden Classification; 6: Anticholinergic Loading Scale; 7: Cancelli's Anticholinergic Burden Scale; 8: Chew's list; 9: Clinical Index and Pharmacological Index; 10: Clinician-rated Anticholinergic Score; 11: Summers' Drug Risk Number; 12: Muscarinic Acetylcholinergic Receptor ANTagonist Exposure Scale; 13: Anticholinergic Effect on Cognition; 14: Anticholinergic Burden Score for German prescribers; 15: Korean Anticholinergic Burden Scale; 16: Anticholinergic Impregnation Scale; 17: Brazilian's scale; 18: Cao's scale; 19: Drug Delirium Scale; 20: Delirogenic Risk Scale; 21: Anticholinergic Toxicity Score; 22: Salahudeen's Composite rating scale; 23: Durán's list																							
Protriptyline	3					3					3							1				1	2
Pyrilamine	3														3							1	2
Pseudoephedrine		2				2								1		2	2					1	
Quetiapine		1	3	2				2	1	2		1	2	2	2	2	2			2		1	1
Quinidine			1								3		1	1		1		1				1	
Ranitidine	2	1	1	2		1		2		2		1		2	1	1	1		1	2	1,3	1	1
Risperidone		1	1			1			1	1		1		1	1	1	1				5	1	1
Rotigotine														1									
Secobarbital											3												
Selegiline		1												1		1	1					1	
Sertindole													1										
Sertraline	1								1	1			1	1		1	1			1		1	
Scopolamine	3		3							3	3			3	3	3			2	4		1	2
Scopolia extract															3								
Sodium Salicylate											2												
Solifenacin			3										1	3	3	3	3		1			1	
Sumatriptan						1								1								1	
Tapentadol																			1				
Temazepam	1					1		2					1	1	1	1			1	2		1	1

Drug	1	2	3	4	5	6	7	8	9	10	11	12	13	14	15	16	17	18	19	20	21	22	23
1: Anticholinergic Drug Scale; 2: Anticholinergic Risk Scale; 3: Anticholinergic Cognitive Burden Scale; 4: Anticholinergic Activity Scale; 5: Anticholinergic Burden Classification; 6: Anticholinergic Loading Scale; 7: Cancelli's Anticholinergic Burden Scale; 8: Chew's list; 9: Clinical Index and Pharmacological Index; 10: Clinician-rated Anticholinergic Score; 11: Summers' Drug Risk Number; 12: Muscarinic Acetylcholinergic Receptor ANTagonist Exposure Scale; 13: Anticholinergic Effect on Cognition; 14: Anticholinergic Burden Score for German prescribers; 15: Korean Anticholinergic Burden Scale; 16: Anticholinergic Impregnation Scale; 17: Brazilian's scale; 18: Cao's scale; 19: Drug Delirium Scale; 20: Delirogenic Risk Scale; 21: Anticholinergic Toxicity Score; 22: Salahudeen's Composite rating scale; 23: Durán's list																							
Terfenadine																		1					
Tetrazepam																				1			
Theophylline	1		1	2	2	2	3					1		2	1	1	1			2		1	1
Tiemonium															3								
Timepidium															3								
Tiquizium															3								
Thiopental											3												
Thioridazine	3	3	3	4				4	1	3	3			3	3		3			4	5	1	2
Tiotixene	1	3							1						1				1			1	2
Tiotropium														1			3						
Tizanidine		3										2		3	2	3	3		1			1	2
Tolterodine	3	2	3			3		4		3		2	2	3	3	3	3		1	4		1	2
Topiramate								1												1			
Tramadol	1					2				2		1		2	2	1	1		1	1		1	1
Trandolapril										1				1								1	
Tanylcypromine																			1				
Trazodone		1	1							1		1		1	1	1	1		1			1	1
Triamcinolone	1													1		1	1			1		1	
Triamterene	1		1											1		1	1			1		1	

Drug	1	2	3	4	5	6	7	8	9	10	11	12	13	14	15	16	17	18	19	20	21	22	23
1: Anticholinergic Drug Scale; 2: Anticholinergic Risk Scale; 3: Anticholinergic Cognitive Burden Scale; 4: Anticholinergic Activity Scale; 5: Anticholinergic Burden Classification; 6: Anticholinergic Loading Scale; 7: Cancelli's Anticholinergic Burden Scale; 8: Chew's list; 9: Clinical Index and Pharmacological Index; 10: Clinician-rated Anticholinergic Score; 11: Summers' Drug Risk Number; 12: Muscarinic Acetylcholinergic Receptor ANTagonist Exposure Scale; 13: Anticholinergic Effect on Cognition; 14: Anticholinergic Burden Score for German prescribers; 15: Korean Anticholinergic Burden Scale; 16: Anticholinergic Impregnation Scale; 17: Brazilian's scale; 18: Cao's scale; 19: Drug Delirium Scale; 20: Delirogenic Risk Scale; 21: Anticholinergic Toxicity Score; 22: Salahudeen's Composite rating scale; 23: Durán's list																							
Triazolam	1									1		1		1	1				1	1		1	1
Trihexyphenidyl	3		3	4	3				1	3	3	2	3	3	3	3	3		2	4	5	1	2
Trifluoperazine	1	3	3						1		3		2				3		1		3,83	1	
Trimebutine															1								
Trimethobenzamide											2												
Trimipramine	3		3	4	3								3	3		3			2	4		1	2
Triprolidine															2	2	3			1			
Tropatepine					3											3						1	2
Trospium chloride			3											3	3	3			1			1	
Valethamate bromide															3								
Valproic acid	1													1		1	1			1		1	
Vancomycin	1													1		1	1			1		1	
Venlafaxine			1			1				1				1	1		1					1	
Warfarine	1		1				2							1		1	1			1		1	
Zaleplon																				1			
Ziprasidone		1												1	1							1	
Zolmitriptan						1								1								1	
Zolpidem																			1	1			

Drug	1	2	3	4	5	6	7	8	9	10	11	12	13	14	15	16	17	18	19	20	21	22	23
1: Anticholinergic Drug Scale; 2: Anticholinergic Risk Scale; 3: Anticholinergic Cognitive Burden Scale; 4: Anticholinergic Activity Scale; 5: Anticholinergic Burden Classification; 6: Anticholinergic Loading Scale; 7: Cancelli's Anticholinergic Burden Scale; 8: Chew's list; 9: Clinical Index and Pharmacological Index; 10: Clinician-rated Anticholinergic Score; 11: Summers' Drug Risk Number; 12: Muscarinic Acetylcholinergic Receptor ANTagonist Exposure Scale; 13: Anticholinergic Effect on Cognition; 14: Anticholinergic Burden Score for German prescribers; 15: Korean Anticholinergic Burden Scale; 16: Anticholinergic Impregnation Scale; 17: Brazilian's scale; 18: Cao's scale; 19: Drug Delirium Scale; 20: Delirogenic Risk Scale; 21: Anticholinergic Toxicity Score; 22: Salahudeen's Composite rating scale; 23: Durán's list																							
Zopiclone																			1	1			
Zotepine															2								
Zuclopenthixol															2								
Atropine (ophthalmic)											3												
Ciylopentolate (ophthalmic)											3												
Dexamethasone (ophthalmic)											2												
Isoprotenenol (ophthalmic)											2												
Phenylephedrine (ophthalmic)											2												
Propranolol (ophthalmic)											2												
Tropicamide (ophthalmic)											3												
