# Supplementary material for: A Universal Pharmacological-Based List of Drugs with Anticholinergic Activity
Source: Pharmaceutics. 2023 Jan 10;15(1):230. doi: 10.3390/pharmaceutics15010230 (PMC9863833; doi:10.3390/pharmaceutics15010230)
Supplement: Supplementary file 1 [file pharmaceutics-15-00230-s001.zip › pharmaceutics-2094031-supplementary/Supplementary Table S2.pdf]

Table S2 - Reported affinity of the 304 drugs for muscarinic receptors subtypes according to each pharmacological database.

Drug	Number of tools	*DrugBank*	*Guide to Pharmacology*	*PDSP Ki database*	*Inxight:Drugs*
Aceprometazine	3	1- 2- 3- 4- 5-			1- 2- 3- 4- 5-
Acepromazine	3	1+ 2+ 3- 4- 5-			1- 2- 3- 4- 5-
Acetazolamide	1	1- 2- 3- 4- 5-	1- 2- 3- 4- 5-	1- 2- 3- 4- 5-	1- 2- 3- 4- 5-
Acetylsalicylic acid	1	1- 2- 3- 4- 5-	1- 2- 3- 4- 5-	1- 2+ 3- 4- 5+	1- 2- 3- 4- 5-
Aclidinium	1	1+ 2+ 3+ 4+ 5+	1+ 2+ 3+ 4+ 5+		1+ 2+ 3- 4+ 5+
Aciclovir	1	1- 2- 3- 4- 5-	1- 2- 3- 4- 5-		1- 2- 3- 4- 5-
Alimemazine	7	1- 2- 3- 4- 5-	1? 2? 3? 4? 5?		1- 2- 3- 4- 5-
Alprazolam	13	1- 2- 3- 4- 5-	1- 2- 3- 4- 5-	1- 2- 3- 4- 5-	1- 2- 3- 4- 5-
Alverine	4	1- 2- 3- 4- 5-			1- 2- 3- 4- 5-
Amiodarone	1	1- 2- 3- 4- 5-	1- 2- 3- 4- 5-	1- 2- 3- 4- 5-	1- 2- 3- 4- 5-
Amitriptyline	23	1+ 2+ 3+ 4+ 5+	1+ 2+ 3+ 4+ 5+	1+ 2+ 3+ 4+ 5+	1- 2- 3- 4- 5-
Amantadine	14	1- 2- 3- 4- 5-	1- 2- 3- 4- 5-	1- 2- 3- 4- 5-	1- 2- 3- 4- 5-
Amphotericin	1	1- 2- 3- 4- 5-			1- 2- 3- 4- 5-
Amisulpride	1	1- 2- 3- 4- 5-	1- 2- 3- 4- 5-	1+ 2+ 3+ 4+ 5+	1- 2- 3- 4- 5-
Amoxapine	6	1+ 2+ 3+ 4+ 5+	1- 2- 3- 4- 5-	1+ 2+ 3+ 4+ 5+	1- 2- 3- 4- 5-
Amoxicillin	2	1- 2- 3- 4- 5-	1- 2- 3- 4- 5-		1- 2- 3- 4- 5-
Ampicillin	6	1- 2- 3- 4- 5-	1- 2- 3- 4- 5-		1- 2- 3- 4- 5-
Amobarbital	1	1- 2- 3- 4- 5-			1- 2- 3- 4- 5-
Aripiprazol	5	1+ 2+ 3+ 4+ 5+	1- 2- 3- 4- 5-	1+ 2+ 3+ 4+ 5+	1- 2- 3- 4- 5-
Asenapine	3	1- 2- 3- 4- 5-	1- 2- 3- 4- 5-		1- 2- 3- 4- 5-
Astemizole	1	1- 2- 3- 4- 5-	1- 2- 3- 4- 5-	1- 2- 3- 4- 5-	1- 2- 3- 4- 5-
Atenolol	6	1- 2- 3- 4- 5-	1- 2- 3- 4- 5-	1- 2- 3- 4- 5-	1- 2- 3- 4- 5-
Atropine	18	1+ 2+ 3+ 4+ 5+	1+ 2+ 3+ 4+ 5+	1+ 2+ 3+ 4+ 5+	1+ 2+ 3+ 4- 5-
Azathioprine	5	1- 2- 3- 4- 5-	1- 2- 3- 4- 5-		1- 2- 3- 4- 5-
Baclofen	10	1- 2- 3- 4- 5-	1- 2- 3- 4- 5-	1- 2- 3- 4- 5-	1- 2- 3- 4- 5-
Belladona	7	1+ 2+ 3+ 4+ 5+			
Benazepril	3	1- 2- 3- 4- 5-	1- 2- 3- 4- 5-		1- 2- 3- 4- 5-
Benzatropine	13	1+ 2- 3- 4- 5-	1+ 2+ 3- 4- 5-	1+ 2+ 3+ 4+ 5+	1+ 2- 3- 4- 5-
Betaxolol	3	1- 2- 3- 4- 5-	1- 2- 3- 4- 5-	1- 2- 3- 4- 5-	1- 2- 3- 4- 5-
Biperiden	7	1+ 2- 3- 4- 5-	1+ 2+ 3+ 4+ 5+	1+ 2+ 3+ 4+ 5-	1+ 2- 3- 4- 5-
Bisacodyl	3	1- 2- 3- 4- 5-			1- 2- 3- 4- 5-
Blonanserin	1	1? 2? 3? 4? 5?	1- 2- 3- 4- 5-		1- 2- 3- 4- 5-
Bromazepam	2	1- 2- 3- 4- 5-			1- 2- 3- 4- 5-
Bromocriptine	7	1- 2- 3- 4- 5-	1- 2- 3- 4- 5-	1- 2- 3- 4- 5-	1- 2- 3- 4- 5-
Brompheniramine	8	1+ 2+ 3+ 4+ 5+	1? 2? 3? 4? 5?	1- 2- 3- 4- 5-	1? 2? 3? 4? 5?
Bromperidol	1	1- 2- 3- 4- 5-		1+ 2+ 3+ 4+ 5+	1- 2- 3- 4- 5-

Drug	Number of tools	*DrugBank*	*Guide to Pharmacology*	*PDSP Ki database*	*Inxight:Drugs*
Brotizolam	1	1- 2- 3- 4- 5-		1- 2- 3- 4- 5-	1- 2- 3- 4- 5-
Buclizine	1	1+ 2- 3- 4- 5-	1? 2? 3? 4? 5?		1? 2? 3? 4? 5?
Buprenorphine	1	1- 2- 3- 4- 5-	1- 2- 3- 4- 5-	1- 2- 3- 4- 5-	1- 2- 3- 4- 5-
Bupropion	8	1- 2- 3- 4- 5-		1+ 2+ 3+ 4+ 5+	1- 2- 3- 4- 5-
Butylscopolamine	2	1- 2+ 3+ 4- 5-			1+ 2+ 3+ 4- 5-
Captopril	7	1- 2- 3- 4- 5-	1- 2- 3- 4- 5-	1- 2- 3- 4- 5-	1- 2- 3- 4- 5-
Carbamazepine	13	1- 2- 3- 4- 5-	1- 2- 3- 4- 5-	1- 2- 3- 4- 5-	1- 2- 3- 4- 5-
Carbidopa-levodopa	7	1- 2- 3- 4- 5-	1- 2- 3- 4- 5-		1- 2- 3- 4- 5-
Carbinoxamine	7	1? 2? 3? 4? 5?	1- 2- 3- 4- 5-	1- 2- 3- 4- 5-	1? 2? 3? 4? 5?
Carisoprodol	3	1- 2- 3- 4- 5-	1- 2- 3- 4- 5-		1- 2- 3- 4- 5-
Cefamandole	2	1- 2- 3- 4- 5-			1- 2- 3- 4- 5-
Cefoxitin	4	1- 2- 3- 4- 5-	1- 2- 3- 4- 5-		1- 2- 3- 4- 5-
Celecoxib	5	1- 2- 3- 4- 5-	1- 2- 3- 4- 5-	1- 2+ 3- 4- 5+	1- 2- 3- 4- 5-
Cefalotin	2	1- 2- 3- 4- 5-	1- 2- 3- 4- 5-		1- 2- 3- 4- 5-
Cefalexin	2	1- 2- 3- 4- 5-	1- 2- 3- 4- 5-		1- 2- 3- 4- 5-
Cetirizine	11	1- 2- 3- 4- 5-	1- 2- 3- 4- 5-	1- 2- 3- 4- 5-	1- 2- 3- 4- 5-
Chlordiazepoxide	11	1- 2- 3- 4- 5-	1- 2- 3- 4- 5-	1- 2- 3- 4- 5-	1- 2- 3- 4- 5-
Chlorohydrate	1				
Chlorpheniramine	14	1- 2- 3- 4- 5-	1- 2- 3- 4- 5-	1- 2- 3- 4- 5-	1- 2- 3- 4- 5-
Chlorphenamine	5				
Chlorpromazine	16	1+ 2- 3+ 4- 5-	1? 2? 3? 4? 5?	1+ 2+ 3+ 4+ 5+	1+ 2+ 3- 4- 5-
Chlortalidone	6	1- 2- 3- 4- 5-	1- 2- 3- 4- 5-		1- 2- 3- 4- 5-
Chlorprothixene	1	1+ 2+ 3+ 4+ 5+		1+ 2+ 3+ 4+ 5+	1+ 2+ 3+ 4+ 5+
Chlorzoxazone	1	1- 2- 3- 4- 5-	1- 2- 3- 4- 5-		1- 2- 3- 4- 5-
Cimetidine	12	1- 2- 3- 4- 5-	1- 2- 3- 4- 5-	1- 2- 3- 4- 5-	1- 2- 3- 4- 5-
Cimetropium	1	1? 2? 3? 4? 5?			1+ 2+ 3+ 4+ 5+
Cinnarizine	1	1+ 2+ 3+ 4+ 5+	1? 2? 3? 4? 5?	1- 2- 3- 4- 5-	1- 2- 3- 4- 5-
Citalopram	13	1- 2- 3- 4- 5-	1- 2- 3- 4- 5-	1+ 2- 3- 4- 5-	1- 2- 3- 4- 5-
Clemastine	10	1? 2? 3? 4? 5?	1- 2- 3- 4- 5-	1- 2- 3- 4- 5-	1? 2? 3? 4? 5?
Clidinium	4	1+ 2- 3- 4- 5-	1+ 2- 3+ 4- 5-		1- 2- 3+ 4- 5-
Clindamycin	5	1- 2- 3- 4- 5-	1- 2- 3- 4- 5-		1- 2- 3- 4- 5-
Clobazam	1	1- 2- 3- 4- 5-	1- 2- 3- 4- 5-	1- 2- 3- 4- 5-	1- 2- 3- 4- 5-
Clomipramine	13	1? 2? 3? 4? 5?	1- 2- 3- 4- 5-	1+ 2+ 3+ 4+ 5+	1- 2- 3- 4- 5-
Clonazepam	11	1- 2- 3- 4- 5-	1- 2- 3- 4- 5-	1- 2- 3- 4- 5-	1- 2- 3- 4- 5-
Clonidine	3	1- 2- 3- 4- 5-	1- 2- 3- 4- 5-	1- 2- 3- 4- 5-	1- 2- 3- 4- 5-
Cloperastine	1	1- 2- 3- 4- 5-			1? 2? 3? 4? 5?
Clorazepate	10	1- 2- 3- 4- 5-	1- 2- 3- 4- 5-		1- 2- 3- 4- 5-
Clotiapine	1			1- 2- 3- 4- 5-	1- 2- 3- 4- 5-

Drug	Number of tools	*DrugBank*	*Guide to Pharmacology*	*PDSP Ki database*	*Inxight:Drugs*
Clozapine	16	1+ 2+ 3+ 4+ 5+	1+ 2- 3- 4- 5-	1+ 2+ 3+ 4+ 5+	1- 2- 3- 4- 5-
Codeine	15	1- 2- 3- 4- 5-	1- 2- 3- 4- 5-	1- 2- 3- 4- 5-	1- 2- 3- 4- 5-
Colchicine	6	1- 2- 3- 4- 5-	1- 2- 3- 4- 5-		1- 2- 3- 4- 5-
Cortisone	3		1- 2- 3- 4- 5-		1- 2- 3- 4- 5-
Cyamemazine	1		1- 2- 3- 4- 5-	1+ 2+ 3+ 4+ 5+	1- 2- 3- 4- 5-
Cyclobenzaprine	9	1- 2- 3- 4- 5-	1- 2- 3- 4- 5-	1+ 2+ 3+ 4- 5-	1- 2- 3- 4- 5-
Cyproheptadine	13	1+ 2+ 3+ 4- 5-	1- 2- 3- 4- 5-		1+ 2+ 3+ 4+ 5+
Cycloserine	2	1- 2- 3- 4- 5-	1- 2- 3- 4- 5-		1- 2- 3- 4- 5-
Ciclosporin	5	1- 2- 3- 4- 5-	1- 2- 3- 4- 5-		1- 2- 3- 4- 5-
Darifenacin	7	1+ 2+ 3+ 4+ 5+	1+ 2+ 3+ 4+ 5+	1+ 2+ 3+ 4+ 5+	1- 2- 3+ 4- 5-
Desipramine	12	1+ 2+ 3+ 4+ 5+	1? 2? 3? 4? 5?	1+ 2+ 3+ 4+ 5+	1- 2- 3- 4- 5-
Desloratadine	6	1- 2- 3- 4- 5-	1- 2- 3- 4- 5-		1- 2- 3- 4- 5-
Desvenlafaxine	2	1- 2- 3- 4- 5-	1- 2- 3- 4- 5-	1+ 2+ 3+ 4+ 5+	1- 2- 3- 4- 5-
Dexamethasone	8	1- 2- 3- 4- 5-	1- 2- 3- 4- 5-	1- 2- 3- 4- 5-	1- 2- 3- 4- 5-
Dexbrompheniramine	2	1- 2- 3- 4- 5-	1? 2? 3? 4? 5?		1? 2? 3? 4? 5?
Dexchlorpheniramine	7	1? 2? 3? 4? 5?		1- 2- 3- 4- 5-	1+ 2+ 3+ 4+ 5+
Dextromethorphan	4	1- 2- 3- 4- 5-	1- 2- 3- 4- 5-	1- 2- 3- 4- 5-	1- 2- 3- 4- 5-
Diazepam	18	1- 2- 3- 4- 5-	1- 2- 3- 4- 5-	1- 2- 3- 4- 5-	1- 2- 3- 4- 5-
Difenidol	1	1+ 2+ 3+ 4- 5-	1? 2? 3? 4? 5?		1+ 2+ 3+ 4+ 5+
Difemerine	1				1? 2? 3? 4? 5?
Dicyclomine	11	1+ 2+ 3+ 4- 5-	1+ 2+ 3+ 4+ 5-	1+ 2+ 3+ 4+ 5-	1+ 2- 3+ 4- 5-
Diphenoxylate	1	1- 2- 3- 4- 5-	1- 2- 3- 4- 5-		1- 2- 3- 4- 5-
Digitoxin	5	1- 2- 3- 4- 5-	1- 2- 3- 4- 5-		1- 2- 3- 4- 5-
Digoxin	14	1- 2- 3- 4- 5-	1- 2- 3- 4- 5-		1- 2- 3- 4- 5-
Diltiazem	6	1- 2- 3- 4- 5-	1- 2- 3- 4- 5-	1- 2- 3- 4- 5-	1- 2- 3- 4- 5-
Dimenhydrinate	11	1? 2? 3? 4? 5?			1- 2- 3- 4- 5-
Dimetindene	2	1- 2+ 3- 4- 5-	1- 2- 3- 4- 5-		1? 2? 3? 4? 5?
Diphenhydramine	19	1- 2+ 3- 4- 5-	1? 2? 3? 4? 5?	1+ 2+ 3+ 4+ 5+	1- 2- 3- 4- 5-
Dipyridamole	6	1- 2- 3- 4- 5-	1- 2- 3- 4- 5-	1- 2- 3- 4- 5-	1- 2- 3- 4- 5-
Disopyramide	7	1+ 2+ 3+ 4- 5-	1- 2- 3- 4- 5-		1- 2- 3- 4- 5-
Disulfiram	1	1- 2- 3- 4- 5-			1- 2- 3- 4- 5-
Divalproex sodium	3				
Domperidone	8	1- 2- 3- 4- 5-	1- 2- 3- 4- 5-	1- 2- 3- 4- 5-	1- 2- 3- 4- 5-
Donepezil	2	1- 2- 3- 4- 5-	1- 2- 3- 4- 5-	1- 2- 3- 4- 5-	1- 2- 3- 4- 5-
Dopamine	1	1- 2- 3- 4- 5-	1- 2- 3- 4- 5-	1- 2- 3- 4- 5-	1- 2- 3- 4- 5-
Dosulepin	6	1+ 2+ 3+ 4+ 5+	1+ 2+ 3+ 4+ 5+	1+ 2+ 3+ 4+ 5+	1+ 2+ 3+ 4+ 5+
Doxepin	18	1+ 2+ 3+ 4+ 5+	1- 2- 3- 4- 5-	1+ 2+ 3+ 4+ 5+	1- 2- 3- 4- 5-
Doxylamine	7	1+ 2+ 3+ 4+ 5+	1? 2? 3? 4? 5?		1? 2? 3? 4? 5?

Drug	Number of tools	*DrugBank*	*Guide to Pharmacology*	*PDSP Ki database*	*Inxight:Drugs*
Duloxetine	4	1- 2- 3- 4- 5-	1- 2- 3- 4- 5-	1+ 2+ 3- 4- 5-	1- 2- 3- 4- 5-
Emedastine	1	1- 2- 3- 4- 5-	1- 2- 3- 4- 5-		1- 2- 3- 4- 5-
Emepronium	3				1? 2? 3? 4? 5?
Entacapone	6	1- 2- 3- 4- 5-	1- 2- 3- 4- 5-		1- 2- 3- 4- 5-
Escitalopram	8	1+ 2- 3- 4- 5-	1- 2- 3- 4- 5-	1+ 2- 3- 4- 5-	1- 2- 3- 4- 5-
Estazolam	3	1- 2- 3- 4- 5-	1- 2- 3- 4- 5-		1- 2- 3- 4- 5-
Ethchlorvynol	1	1- 2- 3- 4- 5-	1- 2- 3- 4- 5-		1- 2- 3- 4- 5-
Etoricoxib	1	1- 2- 3- 4- 5-	1- 2- 3- 4- 5-	1+ 2- 3- 4- 5-	1- 2- 3- 4- 5-
Famotidine	5	1- 2- 3- 4- 5-	1- 2- 3- 4- 5-	1- 2- 3- 4- 5-	1- 2- 3- 4- 5-
Fentanyl	15	1- 2- 3- 4- 5-	1- 2- 3- 4- 5-	1- 2- 3- 4- 5-	1- 2- 3- 4- 5-
Fesoterodine	5	1+ 2+ 3+ 4+ 5+	1- 2+ 3+ 4- 5-		1+ 2+ 3+ 4+ 5+
Fexofenadine	7	1- 2- 3- 4- 5-	1- 2- 3- 4- 5-	1- 2- 3- 4- 5-	1- 2- 3- 4- 5-
Flavoxate	8	1+ 2+ 3- 4- 5-	1? 2? 3? 4? 5?		1+ 2+ 3+ 4+ 5+
Fluoxetine	15	1? 2? 3? 4? 5?	1- 2- 3- 4- 5-	1+ 2+ 3+ 4+ 5+	1? 2? 3? 4? 5?
Fluphenazine	12	1- 2- 3- 4- 5-	1- 2- 3- 4- 5-	1+ 2+ 3+ 4+ 5+	1- 2- 3- 4- 5-
Flunitrazepam	4	1- 2- 3- 4- 5-	1- 2- 3- 4- 5-	1- 2- 3- 4- 5-	1- 2- 3- 4- 5-
Flupentixol	1	1+ 2- 3- 4- 5-	1- 2- 3- 4- 5-	1- 2- 3- 4- 5-	1- 2- 3- 4- 5-
Flurazepam	7	1- 2- 3- 4- 5-	1- 2- 3- 4- 5-		1- 2- 3- 4- 5-
Fluticasone-salmeterol	2	1- 2- 3- 4- 5-	1- 2- 3- 4- 5-	1- 2- 3- 4- 5-	1- 2- 3- 4- 5-
Fluvoxamine	12	1? 2? 3? 4? 5?	1- 2- 3- 4- 5-	1+ 2- 3- 4- 5-	1- 2- 3- 4- 5-
Furosemide	11	1- 2- 3- 4- 5-	1- 2- 3- 4- 5-		1- 2- 3- 4- 5-
Gabapentin	1	1- 2- 3- 4- 5-	1- 2- 3- 4- 5-		1- 2- 3- 4- 5-
Gentamicin	6	1- 2- 3- 4- 5-	1- 2- 3- 4- 5-		1- 2- 3- 4- 5-
Glycopyrronium/ Glycopyrrolate	3	1+ 2+ 3+ 4+ 5+	1+ 2+ 3+ 4+ 5+		1+ 2+ 3+ 4+ 5+
Guaifenesin	4	1- 2- 3- 4- 5-	1- 2- 3- 4- 5-		1- 2- 3- 4- 5-
Haloperidol	12	1- 2- 3+ 4- 5-	1? 2? 3? 4? 5?	1+ 2+ 3+ 4+ 5+	1? 2? 3? 4? 5?
Homatropine	4	1+ 2+ 3+ 4+ 5+		1+ 2+ 3+ 4+ 5+	1+ 2+ 3+ 4+ 5+
Homochlorcyclizine	1				1? 2? 3? 4? 5?
Hydralazine	8	1- 2- 3- 4- 5-	1- 2- 3- 4- 5-	1- 2- 3- 4- 5-	1- 2- 3- 4- 5-
Hydrocodone	7	1- 2- 3- 4- 5-	1- 2- 3- 4- 5-	1- 2- 3- 4- 5-	1- 2- 3- 4- 5-
Hydrocortisone	7	1- 2- 3- 4- 5-	1- 2- 3- 4- 5-		1- 2- 3- 4- 5-
Hydromorphone	1	1- 2- 3- 4- 5-	1- 2- 3- 4- 5-	1- 2- 3- 4- 5-	1- 2- 3- 4- 5-
Hydroxyzine	16	1- 2- 3- 4- 5-	1? 2? 3? 4? 5?	1- 2- 3- 4- 5-	1? 2? 3? 4? 5?
Hyoscyamine	11	1+ 2+ 3+ 4+ 5-	1+ 2+ 3+ 4+ 5+		1+ 2+ 3+ 4- 5-
Iloperidone	3	1- 2- 3- 4- 5-	1- 2- 3- 4- 5-	1+ 2+ 3+ 4+ 5+	1- 2- 3- 4- 5-
Imidafenacin	1	1+ 2+ 3+ 4- 5-			1+ 2- 3+ 4- 5-
Imipramine	20	1+ 2+ 3+ 4+ 5+	1- 2+ 3- 4- 5-	1+ 2+ 3+ 4+ 5+	1- 2- 3- 4- 5-

Drug	Number of tools	*DrugBank*	*Guide to Pharmacology*	*PDSP Ki database*	*Inxight:Drugs*
Ipratropium	8	1+ 2+ 3+ 4- 5-	1+ 2+ 3+ 4+ 5+	1+ 2+ 3+ 4+ 5+	1+ 2+ 3+ 4+ 5+
Isosorbide	4				1- 2- 3- 4- 5-
Isosorbide dinitrate	4	1- 2- 3- 4- 5-	1- 2- 3- 4- 5-		1- 2- 3- 4- 5-
Isosorbide mononitrate	3	1- 2- 3- 4- 5-	1- 2- 3- 4- 5-		1- 2- 3- 4- 5-
Ketotifen ophthalmic	2	1- 2- 3- 4- 5-	1- 2- 3- 4- 5-	1- 2- 3- 4- 5-	1- 2- 3- 4- 5-
Ketotifen	1	1- 2- 3- 4- 5-	1- 2- 3- 4- 5-	1- 2- 3- 4- 5-	1- 2- 3- 4- 5-
Ketorolac	4	1- 2- 3- 4- 5-	1- 2- 3- 4- 5-		1- 2- 3- 4- 5-
Lansoprazole	4	1- 2- 3- 4- 5-	1- 2- 3- 4- 5-		1- 2- 3- 4- 5-
Levocetirizine	5	1- 2- 3- 4- 5-	1- 2- 3- 4- 5-	1- 2- 3- 4- 5-	1- 2- 3- 4- 5-
Levofloxacin	2	1- 2- 3- 4- 5-	1- 2- 3- 4- 5-		1- 2- 3- 4- 5-
Levomepromazine	12	1+ 2+ 3+ 4+ 5+	1? 2? 3? 4? 5?	1- 2- 3- 4- 5-	1- 2- 3- 4- 5-
Lidocaine	1	1- 2- 3- 4- 5-	1- 2- 3- 4- 5-		1- 2- 3- 4- 5-
Lithium	10	1- 2- 3- 4- 5-	1- 2- 3- 4- 5-		1- 2- 3- 4- 5-
Lofepramine	1		1? 2? 3? 4? 5?	1+ 2+ 3+ 4+ 5+	1+ 2+ 3+ 4+ 5+
Loperamide	13	1- 2- 3- 4- 5-	1- 2- 3- 4- 5-	1- 2- 3- 4- 5-	1- 2- 3- 4- 5-
Loratadine	11	1- 2- 3- 4- 5-	1- 2- 3- 4- 5-		1? 2? 3? 4? 5?
Lorazepam	8	1- 2- 3- 4- 5-	1- 2- 3- 4- 5-		1- 2- 3- 4- 5-
Lormetazepam	1	1- 2- 3- 4- 5-	1- 2- 3- 4- 5-		1- 2- 3- 4- 5-
Loxapine	9	1+ 2+ 3+ 4+ 5+	1- 2- 3- 4- 5-	1+ 2+ 3+ 4+ 5+	1+ 2- 3- 4- 5-
Lumiracoxib	2	1- 2- 3- 4- 5-	1- 2- 3- 4- 5-		1- 2- 3- 4- 5-
Maprotiline	6	1+ 2+ 3+ 4+ 5+	1? 2? 3? 4? 5?	1- 2- 3- 4- 5-	1? 2? 3? 4? 5?
Mebeverine	1		1? 2? 3? 4? 5?		1+ 2+ 3+ 4+ 5+
Meclizine	8	1? 2? 3? 4? 5?	1? 2? 3? 4? 5?	1- 2- 3- 4- 5-	1? 2? 3? 4? 5?
Medazepam	1	1- 2- 3- 4- 5-			1- 2- 3- 4- 5-
Mequitazine	2	1- 2- 3- 4- 5-		1+ 2+ 3+ 4+ 5+	1+ 2+ 3+ 4- 5-
Mesoridazine	1	1- 2- 3- 4- 5-	1- 2- 3- 4- 5-	1+ 2+ 3+ 4+ 5+	1- 2- 3- 4- 5-
Metaraminol	1	1- 2- 3- 4- 5-	1- 2- 3- 4- 5-		1- 2- 3- 4- 5-
Metformin	5	1- 2- 3- 4- 5-	1- 2- 3- 4- 5-		1- 2- 3- 4- 5-
Mehadone	6	1- 2- 3- 4- 5-	1- 2- 3- 4- 5-	1- 2- 3- 4- 5-	1- 2- 3- 4- 5-
Methocarbamol	9	1- 2- 3- 4- 5-	1- 2- 3- 4- 5-		1- 2- 3- 4- 5-
Methotrexate	3	1- 2- 3- 4- 5-	1- 2- 3- 4- 5-		1- 2- 3- 4- 5-
Methohexital	1	1- 2- 3- 4- 5-	1- 2- 3- 4- 5-		1- 2- 3- 4- 5-
Methyldopa	1	1- 2- 3- 4- 5-	1- 2- 3- 4- 5-		1- 2- 3- 4- 5-
Methylprednisolone	7	1- 2- 3- 4- 5-	1- 2- 3- 4- 5-		1- 2- 3- 4- 5-
Metoclopramide	7	1+ 2- 3- 4- 5-	1? 2? 3? 4? 5?	1+ 2+ 3+ 4+ 5+	1+ 2- 3+ 4- 5-
Metoprolol	6	1- 2- 3- 4- 5-	1- 2- 3- 4- 5-	1- 2- 3- 4- 5-	1- 2- 3- 4- 5-
Midazolam	7	1- 2- 3- 4- 5-	1- 2- 3- 4- 5-	1- 2- 3- 4- 5-	1- 2- 3- 4- 5-
Mirtazapine	12	1- 2- 3- 4- 5-	1- 2- 3- 4- 5-	1+ 2+ 3+ 4+ 5+	1- 2- 3- 4- 5-

Drug	Number of tools	*DrugBank*	*Guide to Pharmacology*	*PDSP Ki database*	*Inxight:Drugs*
Moclobemide	1	1? 2? 3? 4? 5?	1- 2- 3- 4- 5-		1- 2- 3- 4- 5-
Molindone	5	1+ 2- 3- 4- 5-	1- 2- 3- 4- 5-	1+ 2+ 3+ 4- 5-	1- 2- 3- 4- 5-
Morphine	13	1- 2- 3- 4- 5-	1- 2- 3- 4- 5-	1- 2- 3- 4- 5-	1- 2- 3- 4- 5-
Naratriptan	3	1- 2- 3- 4- 5-	1- 2- 3- 4- 5-	1- 2- 3- 4- 5-	1- 2- 3- 4- 5-
Nefopam	3			1- 2- 3- 4- 5-	1- 2- 3- 4- 5-
Nefazodone	3	1- 2- 3- 4- 5-	1- 2- 3- 4- 5-	1+ 2+ 3+ 4+ 5+	1- 2- 3- 4- 5-
Nifedipine	8	1- 2- 3- 4- 5-	1- 2- 3- 4- 5-	1- 2- 3- 4- 5-	1- 2- 3- 4- 5-
Nitrazepam	2	1- 2- 3- 4- 5-		1- 2- 3- 4- 5-	1- 2- 3- 4- 5-
Nitroprusside	1	1- 2- 3- 4- 5-	1- 2- 3- 4- 5-		1- 2- 3- 4- 5-
Nizatidine	3	1- 2- 3- 4- 5-	1- 2- 3- 4- 5-		1- 2- 3- 4- 5-
Nortriptyline	19	1+ 2+ 3+ 4+ 5+	1? 2? 3? 4? 5?	1- 2- 3- 4- 5-	1- 2- 3- 4- 5-
Octylonium bromide	1				1+ 2+ 3+ 4+ 5-
Olanzapine	18	1+ 2+ 3+ 4+ 5-	1? 2? 3? 4? 5?	1+ 2+ 3+ 4+ 5+	1? 2? 3? 4? 5?
Opipramol	4		1- 2- 3- 4- 5-	1- 2- 3- 4- 5-	1? 2? 3? 4? 5?
Orphenadrine	13	1? 2? 3? 4? 5?	1? 2? 3? 4? 5?	1+ 2+ 3+ 4+ 5+	1+ 2+ 3+ 4+ 5+
Oxapium iodide	1				1+ 2+ 3+ 4+ 5+
Oxazepam	7	1- 2- 3- 4- 5-	1- 2- 3- 4- 5-	1- 2- 3- 4- 5-	1- 2- 3- 4- 5-
Oxcarbazepine	10	1- 2- 3- 4- 5-	1- 2- 3- 4- 5-		1- 2- 3- 4- 5-
Oxitropium bromide	1	1? 2? 3? 4? 5?			1+ 2+ 3+ 4+ 5+
Oxybutynin	19	1+ 2+ 3+ 4- 5-	1+ 2+ 3+ 4+ 5+	1+ 2+ 3+ 4- 5-	1+ 2+ 3+ 4- 5-
Oxycodone	12	1- 2- 3- 4- 5-	1- 2- 3- 4- 5-	1- 2- 3- 4- 5-	1- 2- 3- 4- 5-
Paliperidone	4	1- 2- 3- 4- 5-	1- 2- 3- 4- 5-		1- 2- 3- 4- 5-
Pancuronium	3	1- 2+ 3+ 4- 5-	1- 2- 3- 4- 5-	1- 2- 3- 4- 5-	1- 2- 3- 4- 5-
Paroxetine	18	1+ 2+ 3+ 4+ 5+	1- 2- 3- 4- 5-	1+ 2+ 3+ 4+ 5+	1- 2- 3- 4- 5-
Pentazocine	1	1- 2- 3- 4- 5-	1- 2- 3- 4- 5-	1- 2- 3- 4- 5-	1- 2- 3- 4- 5-
Perphenazine	15	1- 2- 3- 4- 5-	1- 2- 3- 4- 5-	1+ 2+ 3+ 4- 5-	1+ 2- 3- 4- 5-
Pethidine	12	1+ 2+ 3+ 4+ 5+	1- 2- 3- 4- 5-		1- 2- 3- 4- 5-
Phenelzine	5	1- 2- 3- 4- 5-	1- 2- 3- 4- 5-	1- 2- 3- 4- 5-	1- 2- 3- 4- 5-
Pheniramine	1	1- 2- 3- 4- 5-	1- 2- 3- 4- 5-	1- 2- 3- 4- 5-	1- 2- 3- 4- 5-
Phenytoin	3	1- 2- 3- 4- 5-	1- 2- 3- 4- 5-		1- 2- 3- 4- 5-
Phenobarbital	7	1- 2- 3- 4- 5-	1- 2- 3- 4- 5-		1- 2- 3- 4- 5-
Pimozide	12	1- 2- 3- 4- 5-	1- 2- 3- 4- 5-	1+ 2+ 3+ 4+ 5+	1- 2- 3- 4- 5-
Piperacillin	5	1- 2- 3- 4- 5-	1- 2- 3- 4- 5-		1- 2- 3- 4- 5-
Pipotiazine	1	1? 2? 3? 4? 5?	1? 2? 3? 4? 5?	1- 2- 3- 4- 5-	1- 2- 3- 4- 5-
Piprinhydrinate	1	1? 2? 3? 4? 5?			1? 2? 3? 4? 5?
Pramipexole	6	1- 2- 3- 4- 5-	1- 2- 3- 4- 5-	1+ 2+ 3+ 4+ 5+	1- 2- 3- 4- 5-
Prazepam	1	1- 2- 3- 4- 5-	1- 2- 3- 4- 5-		1- 2- 3- 4- 5-
Prednisolone	7	1- 2- 3- 4- 5-	1- 2- 3- 4- 5-	1- 2- 3- 4- 5-	1- 2- 3- 4- 5-

Drug	Number of tools	*DrugBank*	*Guide to Pharmacology*	*PDSP Ki database*	*Inxight:Drugs*
Prednisone	9	1- 2- 3- 4- 5-	1- 2- 3- 4- 5-	1- 2- 3- 4- 5-	1- 2- 3- 4- 5-
Pregabalin	1	1- 2- 3- 4- 5-	1- 2- 3- 4- 5-		1- 2- 3- 4- 5-
Pridinol	1				1+ 2+ 3+ 4+ 5+
Procainamide	1	1- 2- 3- 4- 5-	1- 2- 3- 4- 5-	1- 2+ 3- 4- 5-	1- 2- 3- 4- 5-
Procyclidine	8	1+ 2+ 3+ 4+ 5-	1? 2? 3? 4? 5?	1+ 2+ 3+ 4+ 5+	1+ 2+ 3+ 4+ 5-
Prochlorperazine	11	1- 2- 3- 4- 5-	1- 2- 3- 4- 5-	1+ 2+ 3+ 4+ 5+	1- 2- 3- 4- 5-
Promazine	6	1+ 2- 3- 4- 5-	1- 2- 3- 4- 5-	1+ 2+ 3+ 4+ 5+	1? 2? 3? 4? 5?
Prometazine	15	1+ 2+ 3+ 4+ 5+	1? 2? 3? 4? 5?	1- 2- 3- 4- 5-	1? 2? 3? 4? 5?
Propantheline	7	1+ 2- 3- 4- 5-	1+ 2+ 3+ 4+ 5-		1+ 2+ 3+ 4+ 5-
Periciazine	1	1? 2? 3? 4? 5?	1? 2? 3? 4? 5?		1? 2? 3? 4? 5?
Propiverine	4	1+ 2+ 3+ 4+ 5+			1+ 2+ 3+ 4+ 5+
Propoxyphene	6	1- 2- 3- 4- 5-	1- 2- 3- 4- 5-		1- 2- 3- 4- 5-
Propranolol	1	1- 2- 3- 4- 5-	1- 2- 3- 4- 5-	1- 2- 3- 4- 5-	1- 2- 3- 4- 5-
Protriptyline	6	1? 2? 3? 4? 5?	1- 2- 3- 4- 5-	1+ 2+ 3+ 4+ 5+	1? 2? 3? 4? 5?
Pyrilamine	4	1- 2- 3- 4- 5-	1- 2- 3- 4- 5-	1- 2- 3- 4- 5-	1- 2- 3- 4- 5-
Pseudoephedrine	6	1- 2- 3- 4- 5-	1- 2- 3- 4- 5-	1- 2- 3- 4- 5-	1- 2- 3- 4- 5-
Quetiapine	15	1+ 2+ 3+ 4+ 5+	1- 2- 3- 4- 5-	1+ 2+ 3+ 4+ 5+	1? 2? 3? 4? 5?
Quinidine	7	1? 2? 3? 4? 5?	1- 2- 3- 4- 5-	1- 2+ 3- 4- 5-	1- 2- 3- 4- 5-
Ranitidine	17	1? 2? 3? 4? 5?	1- 2- 3- 4- 5-	1- 2- 3- 4- 5-	1- 2- 3- 4- 5-
Risperidone	13	1- 2- 3- 4- 5-	1- 2- 3- 4- 5-	1+ 2+ 3+ 4+ 5+	1- 2- 3- 4- 5-
Rotigotine	1	1- 2- 3- 4- 5-	1- 2- 3- 4- 5-		1- 2- 3- 4- 5-
Secobarbital	1	1- 2- 3- 4- 5-	1- 2- 3- 4- 5-		1- 2- 3- 4- 5-
Selegiline	5	1- 2- 3- 4- 5-	1- 2- 3- 4- 5-		1- 2- 3- 4- 5-
Sertindole	1	1- 2- 3- 4- 5-	1- 2- 3- 4- 5-	1+ 2+ 3+ 4+ 5+	1- 2- 3- 4- 5-
Sertraline	9	1? 2? 3? 4? 5?	1- 2- 3- 4- 5-	1+ 2+ 3+ 4+ 5+	1- 2- 3- 4- 5-
Scopolamine	11	1+ 2+ 3+ 4+ 5+	1+ 2+ 3+ 4+ 5+	1+ 2+ 3+ 4+ 5+	1+ 2+ 3+ 4+ 5+
Scopolia extract	1				
Sodium Salicylate	1	1- 2- 3- 4- 5-		1- 2- 3- 4- 5-	1- 2- 3- 4- 5-
Solifenacin	8	1+ 2+ 3+ 4+ 5+	1+ 2+ 3+ 4+ 5+		1+ 2+ 3+ 4+ 5+
Sumatriptan	3	1- 2- 3- 4- 5-	1- 2- 3- 4- 5-	1- 2- 3- 4- 5-	1- 2- 3- 4- 5-
Tapentadol	1	1- 2- 3- 4- 5-	1- 2- 3- 4- 5-	1- 2- 3- 4- 5-	1- 2- 3- 4- 5-
Temazepam	11	1- 2- 3- 4- 5-	1- 2- 3- 4- 5-		1- 2- 3- 4- 5-
Terfenadine	1	1+ 2+ 3+ 4+ 5+	1- 2- 3- 4- 5-	1- 2- 3- 4- 5-	1- 2- 3- 4- 5-
Tetrazepam	1				1- 2- 3- 4- 5-
Theophylline	14	1- 2- 3- 4- 5-	1- 2- 3- 4- 5-	1- 2- 3- 4- 5-	1- 2- 3- 4- 5-
Tiemonium	1	1? 2? 3? 4? 5?			1+ 2+ 3+ 4+ 5+
Timepidium	1	1? 2? 3? 4? 5?			1+ 2+ 3+ 4+ 5+
Tiquizium	1				1+ 2+ 3+ 4+ 5+

Drug	Number of tools	*DrugBank*	*Guide to Pharmacology*	*PDSP Ki database*	*Inxight:Drugs*
Thiopental	1	1- 2- 3+ 4- 5-	1- 2- 3- 4- 5-		1- 2- 3- 4- 5-
Thioridazine	15	1? 2? 3? 4? 5?	1- 2- 3- 4- 5-	1+ 2+ 3+ 4+ 5+	1? 2? 3? 4? 5?
Tiotixene	7	1+ 2+ 3- 4- 5-	1? 2? 3? 4? 5?	1+ 2+ 3+ 4- 5-	1- 2- 3- 4- 5-
Tiotropium	2	1+ 2+ 3+ 4+ 5+	1+ 2+ 3+ 4+ 5+		1- 2- 3+ 4- 5-
Tizanidine	9	1- 2- 3- 4- 5-	1- 2- 3- 4- 5-	1- 2- 3- 4- 5-	1- 2- 3- 4- 5-
Tolterodine	16	1+ 2+ 3+ 4+ 5+	1+ 2+ 3+ 4+ 5+		1- 2+ 3+ 4- 5-
Topiramate	2	1- 2- 3- 4- 5-	1- 2- 3- 4- 5-	1- 2- 3- 4- 5-	1- 2- 3- 4- 5-
Tramadol	12	1+ 2- 3+ 4- 5-	1- 2- 3- 4- 5-		1- 2- 3- 4- 5-
Trandolapril	3	1- 2- 3- 4- 5-	1- 2- 3- 4- 5-		1- 2- 3- 4- 5-
Tanylcypromine	1	1- 2- 3- 4- 5-	1- 2- 3- 4- 5-	1- 2- 3- 4- 5-	1- 2- 3- 4- 5-
Trazodone	11	1- 2- 3- 4- 5-	1- 2- 3- 4- 5-	1+ 2+ 3+ 4+ 5+	1- 2- 3- 4- 5-
Triamcinolone	6	1- 2- 3- 4- 5-	1- 2- 3- 4- 5-		1- 2- 3- 4- 5-
Triamterene	7	1- 2- 3- 4- 5-	1- 2- 3- 4- 5-		1- 2- 3- 4- 5-
Triazolam	9	1- 2- 3- 4- 5-	1- 2- 3- 4- 5-	1- 2- 3- 4- 5-	1- 2- 3- 4- 5-
Trihexyphenidyl	18	1+ 2+ 3+ 4+ 5+	1+ 2- 3- 4- 5-	1+ 2+ 3+ 4+ 5+	1+ 2- 3+ 4+ 5+
Trifluoperazine	10	1- 2- 3- 4- 5-	1- 2- 3- 4- 5-	1+ 2+ 3+ 4+ 5+	1? 2? 3? 4? 5?
Trimebutine	1	1+ 2+ 3+ 4+ 5-			1? 2? 3? 4? 5?
Trimethobenzamide	1		1- 2- 3- 4- 5-		1- 2- 3- 4- 5-
Trimipramine	11	1+ 2+ 3+ 4+ 5+	1? 2? 3? 4? 5?	1+ 2+ 3+ 4+ 5+	1- 2- 3- 4- 5-
Triprolidine	4	1? 2? 3? 4? 5?	1- 2- 3- 4- 5-	1- 2- 3- 4- 5-	1- 2- 3- 4- 5-
Tropatepine	4	1? 2? 3? 4? 5?			1? 2? 3? 4? 5?
Trospium chloride	6	1+ 2- 3+ 4- 5-	1? 2? 3? 4? 5?		1+ 2+ 3+ 4+ 5+
Valethamate bromide	1	1? 2? 3? 4? 5?			1+ 2+ 3+ 4- 5-
Valproic acid	6	1- 2- 3- 4- 5-	1- 2- 3- 4- 5-		1- 2- 3- 4- 5-
Vancomycin	6	1- 2- 3- 4- 5-	1- 2- 3- 4- 5-		1- 2- 3- 4- 5-
Venlafaxine	7	1? 2? 3? 4? 5?	1- 2- 3- 4- 5-	1+ 2+ 3+ 4+ 5+	1- 2- 3- 4- 5-
Warfarine	8	1- 2- 3- 4- 5-	1- 2- 3- 4- 5-		1- 2- 3- 4- 5-
Zaleplon	1	1- 2- 3- 4- 5-	1- 2- 3- 4- 5-	1- 2- 3- 4- 5-	1- 2- 3- 4- 5-
Ziprasidone	4	1+ 2+ 3+ 4+ 5+	1- 2- 3- 4- 5-	1+ 2+ 3+ 4+ 5+	1- 2- 3- 4- 5-
Zolmitriptan	3	1- 2- 3- 4- 5-	1- 2- 3- 4- 5-		1- 2- 3- 4- 5-
Zolpidem	2	1- 2- 3- 4- 5-	1- 2- 3- 4- 5-	1- 2- 3- 4- 5-	1- 2- 3- 4- 5-
Zopiclone	2	1- 2- 3- 4- 5-	1- 2- 3- 4- 5-		1- 2- 3- 4- 5-
Zotepine	1	1- 2- 3- 4- 5-	1- 2- 3- 4- 5-	1+ 2+ 3+ 4+ 5+	1- 2- 3- 4- 5-
Zuclopenthixol	1	1- 2- 3- 4- 5-	1- 2- 3- 4- 5-		1- 2- 3- 4- 5-
Atropine (ophthalmic)	1	1+ 2+ 3+ 4+ 5+	1+ 2+ 3+ 4+ 5+	1+ 2+ 3+ 4+ 5+	1+ 2+ 3+ 4- 5-
Ciylopentolate (ophthalmic)	1	1+ 2- 3- 4- 5-	1? 2? 3? 4? 5?	1+ 2+ 3+ 4+ 5-	1+ 2+ 3+ 4+ 5+
Dexamethasone (ophthalmic)	1	1- 2- 3- 4- 5-	1- 2- 3- 4- 5-	1- 2- 3- 4- 5-	1- 2- 3- 4- 5-

Drug	Number of tools	*DrugBank*	*Guide to Pharmacology*	*PDSP Ki database*	*Inxight:Drugs*
Isoprotenenol (ophthalmic)	1				1- 2- 3- 4- 5-
Phenylephedrine (ophthalmic)	1	1- 2- 3- 4- 5-	1- 2- 3- 4- 5-	1- 2- 3- 4- 5-	1- 2- 3- 4- 5-
Propranolol (ophthalmic)	1	1- 2- 3- 4- 5-	1- 2- 3- 4- 5-	1- 2- 3- 4- 5-	1- 2- 3- 4- 5-
Tropicamide (ophthalmic)	1	1+ 2+ 3+ 4+ 5-	1- 2+ 3+ 4+ 5+	1+ 2+ 3+ 4+ 5-	1- 2- 3+ 4- 5-

1, 2, 3, 4 e 5: muscarinic receptors subtypes M1, M2, M3, M4 e M5

+: reported affinity

-: without reported affinity

?: affinity for muscarinic receptors was recognized in any database but no experimental measure of ligand action was identified
